# Occurrence, Fate, Effects, and Risks of Dexamethasone: Ecological Implications Post-COVID-19

**DOI:** 10.3390/ijerph182111291

**Published:** 2021-10-27

**Authors:** Ndeke Musee, Lemme Prica Kebaabetswe, Shepherd Tichapondwa, Gosaitse Tubatsi, Ntombikayise Mahaye, Samuel Keeng Leareng, Philiswa Nosizo Nomngongo

**Affiliations:** 1 Emerging Contaminants Ecological Risk Assessment (ECERA) Group, Department of Chemical Engineering, University of Pretoria, Pretoria 0002, South Africa; mahaye.ntombi@gmail.com (N.M.); sleareng@gmail.com (S.K.L.); 2 Department of Biological Sciences and Biotechnology, Botswana International University of Science and Technology, Palapye, Botswana; kebaabetswel@biust.ac.bw (L.P.K.); gosatubs@gmail.com (G.T.); 3 Department of Chemical Engineering, Water Utilization and Environmental Engineering Division, University of Pretoria, Pretoria 0002, South Africa; shepherd.tichapondwa@up.ac.za; 4 Department of Science and Innovation (DSI)/National Research Foundation (NRF) South African Research Chair Initiative (SARChI), Nanotechnology for Water, University of Johannesburg, Doornfontein 2028, South Africa; pnnomngongo@uj.ac.za

**Keywords:** surface water, wastewater, anti-inflammatory drug, transformation, glucocorticoid, drinking water

## Abstract

The recent outbreak of respiratory syndrome-coronavirus-2 (SARS-CoV-2), which causes coronavirus disease (COVID-19), has led to the widespread use of therapeutics, including dexamethasone (DEXA). DEXA, a synthetic glucocorticoid, is among the widely administered drugs used to treat hospitalized COVID-19 patients. The global COVID-19 surge in infections, consequent increasing hospitalizations, and other DEXA applications have raised concerns on eminent adverse ecological implications to aquatic ecosystems. Here, we aim to summarize published studies on DEXA occurrence, fate, and effects on organisms in natural and engineered systems as, pre-COVID, the drug has been identified as an emerging environmental contaminant. The results demonstrated a significant reduction of DEXA in wastewater treatment plants, with a small portion, including its transformation products (TPs), being released into downstream waters. Fish and crustaceans are the most susceptible species to DEXA exposure in the parts-per-billion range, suggesting potential deleterious ecological effects. However, there are data deficits on the implications of DEXA to marine and estuarine systems and wildlife. To improve DEXA management, toxicological outcomes of DEXA and formed TPs should entail long-term studies from whole organisms to molecular effects in actual environmental matrices and at realistic exposure concentrations. This can aid in striking a fine balance of saving human lives and protecting ecological integrity.

## 1. Introduction

### 1.1. COVID-19 and Dexamethasone

A new coronavirus disease (COVID-19), caused by severe acute respiratory syndrome-coronavirus-2 (SARS-CoV-2) [1,2], emerged in 2019 from a zoonotic source [3,4] and was subsequently declared a global pandemic by the World Health Organization (WHO) in March 2020 [5]. At the time of declaration, over 120,000 cases and about 4300 deaths had been reported in 110 countries [6]. Compared with the SARS-CoV that caused an outbreak of SARS in 2003 [7], SARS-CoV-2 has exhibited a stronger transmission capacity [8,9]. Hence, by 23 June 2020, infections had risen to 9.1 million, with 472,700 deaths associated with the disease in 188 countries [6] recorded. Further, these infections increased exponentially in a month (4 October 2020) to 35 million COVID-19 cases and over 1,034,000 related deaths worldwide [6], showing a dramatic upward trend.

At present, without therapeutic antivirals to cure COVID-19 infections, several candidate drugs have been proposed to treat patients, including remdesivir, lopinavir, emetine, chloroquine, and dexamethasone, among others, with many at the clinical phase [10,11,12], owing to their anti-inflammatory activities [13]; the drugs are summarized in Table 1. Moreover, synthetic corticosteroids have been recommended to treat severe cases of COVID-19 [8,9,14,15,16], with their treatment use accounting for up to 50% [8]; they have been found to aid the recovery of patients on ventilators and to improve the condition of those receiving oxygen but not on ventilators, with a concomitant 20% death reduction [17]. 

Dexamethasone (DEXA)—a synthetic glucocorticoid—has been reported as the first effective treatment for the sickest patients with COVID-19, and, given its low cost, known safety profile, and widespread availability, it is a likely candidate for immediate worldwide use [9]. DEXA is an anti-inflammatory agent discovered in 1958, widely used as a therapeutic to treat variant diseases in humans, including meningitis, myeloma [18,19], and bronchiolitis [20] as well as in livestock [21,22,23], among others (Table S1). For COVID-19 patients, DEXA has been demonstrated to prolong the analgesic effect as the disease causes diffuse lung damage mediated through pro-inflammatory cytokines. Hence, DEXA is now used to delay respiratory failure and ultimate death in COVID-19 patients [17] by minimizing the damaging effect of cytokines. Therefore, due to the high number of COVID-19 cases worldwide requiring hospitalization and treatment, DEXA’s ability to reduce mortality, as announced by WHO (June 2020), has triggered a dramatic increase in its demand and use. For instance, DEXA has been approved for coronavirus treatment by countries including the USA, the UK, Japan, South Africa, and Taiwan, among other states. Ultimately, increasing emissions of DEXA into ecosystems from sources that include hospitals, households, and manufacturing plants is of increasing global concern. 

### 1.2. Challenges of DEXA in Aquatic Systems

Similar to other active pharmaceutical ingredients (APIs), DEXA has contributed to the treatment of millions globally for various medical conditions, as summarized in Table S1. However, DEXA has been identified among APIs of emerging concern (EC) in the aquatic systems [24,25,26], thanks to advances in analytical techniques with very low detection limits (parts per trillion (ppt) levels). This is because, firstly, DEXA is a known endocrine disruptor as it is in the family of glucocorticoids [27,28,29,30,31,32,33]. Secondly, Bu and colleagues [26] have raised the possibility of DEXA as an EC of concern since it is a high production volume (HPV) drug with consequent large emissions into ecosystems.

Thirdly, DEXA as a hormone medicine can induce deleterious toxicological outcomes at very low doses [34]. Lastly, there is increasing evidence on the ubiquitous presence of DEXA in different environmental matrices, including wastewater, surface water, river water, and drinking water in the ng/L–µg/L range across different regions over the globe, with highly likely increases following its use in response to the COVID-19 pandemic [35,36,37,38].

Moreover, the DEXA presence in surface [39] and drinking waters is indicative evidence of its persistence and the inability of conventional wastewater treatment systems to remove it from wastewater (e.g., activated sludge with a low removal efficiency of 30–40%) [40]. Accordingly, there is the likelihood of eventual entry into portable water treatment plants, though the removal efficiencies in such systems remain unquantified. Thus, besides DEXA being relatively used in low quantities compared to other glucocorticoids (including hydrocortisone and prednisolone; e.g., see reported quantities in China (2253 tons) [26] and the UK (27 kg) [41]), its structural modifications play a Janus-faced role. Therefore, by design, the DEXA drug is stable in patients but, inadvertently, can be resistant to removal using conventional treatment techniques and, in turn, persists in different environmental matrices. Additionally, owing to the continuous release of small quantities from hospitals, households, and livestock, DEXA can be termed a “pseudo-persistent” API. This challenge is further compounded by the lack of half-lives for DEXA under different environmental matrices, especially now, under wide use in response to COVID-19. 

The effects of DEXA on aquatic organisms, especially lower-tier organisms, including bacteria, *D. magna*, and algae, are limited. Available data suggest potential deleterious effects of DEXA on organisms at different levels of organization, including bacteria [42,43], Daphnia magna [44], fish [45,46,47], and algae [44,48]. Moreover, whether their low environmental concentrations can induce acute and chronic effects on humans, through drinking water, for example, remain an open scientific inquiry. Several studies have demonstrated the deleterious effects of glucocorticoids to aquatic organisms, including fish, at environmentally relevant concentrations [49], and, hence, the recent demand and consequent increase in the use of DEXA in response to COVID-19 triggers an urgent necessity for systematic evaluations of its potential impacts to ecosystems.

Herein, we present a critical examination of data on the chemistry, occurrence, fate, ecotoxicological effects, and treatment of DEXA in wastewater treatment plants (WWTPs), including their metabolites and transformation products (TPs), as reported in the current literature. Further, we examine the extent to which drinking water treatment systems can effectively remove DEXA, given the expected use under both the pre- and post-COVID-19 eras as well as plausible human health effects. Additionally, we consider if current levels of DEXA in surface waters can induce potential ecological risks. Lastly, the article closes with highlights on a set of knowledge gaps and recommendations to map the way forward in the pursuit to balance the need to respond to a global pandemic without comprising ecological integrity. 

## 2. Occurrence of DEXA in the Environment 

### 2.1. Sources of DEXA and Its Metabolites 

Subsequent to adsorption, distribution, metabolism, and excretion (ADME) processes, API compounds excreted from humans and livestock may constitute partial or complete conversion of DEXA into variant metabolites. Glucocorticoids, e.g., DEXA, are poorly absorbed by organisms, with excretion rates of 50–90% of the parent drug compounds through urine and/or feces [50,51]. Hence, the DEXA and its metabolites found in the environment are from sources that include domestic sewage, hospital discharges, and industrial effluents, mostly ending up in WWTPs [52].

The major urinary metabolite in humans is 6β-hydroxydexamethasone [53,54]. Other dominant metabolites of DEXA from the human liver, based on in vitro and in vivo analyses, includes 6α-hydroxydexamethasone, 6-hydroxy-9α-fluoro-androsta-1,4-diene-11β-hydroxy-16α-methyl-3,17-dione (6-hydroxy-9α-F-A), and 9α fluoro-androsta-1,4-diene-11β-hydroxy-16α-methyl-3,17-dione (9a-F-A) [53,54,55]. Dumasia et al. [56] investigated the biotransformation of DEXA in castrated male horses, with the key metabolites identified as 6-hydroxydexamethasone, together with 17-oxo-dexamethasone, 20-dihydrodexamethasone, 6-hydroxy-17-oxodexamethasone, and 11-dehydrodexamethasone. Thus, the marked differences of metabolites produced by the horses compared to those from humans, based on spectra results, are strongly dependent on the transformation pathways specific to the organism type in question [53,56].

### 2.2. Presence of DEXA in the Environment

**Figure 1:**
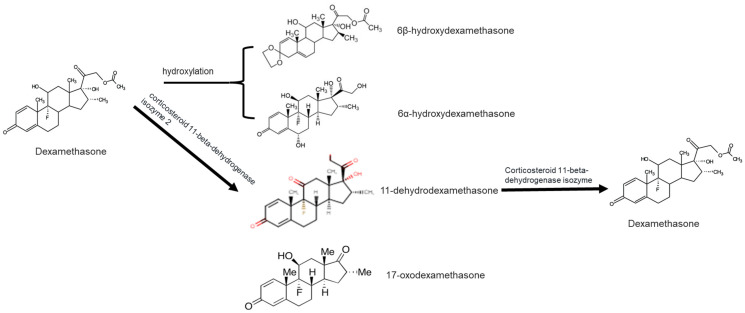
Structure of dexamethasone and formation of likely transformation products based on the dominant process in a given environment.

In the aquatic environment, DEXA and its metabolites may undergo variant transformation processes dependent on biotic and abiotic conditions, as illustrated in Figure 1. These processes may include photo-transformation, hydrolysis, biodegradation, and adsorption, especially in soil and sludge matrices following contact with aqueous media, as observed elsewhere for other forms of APIs [57]. For example, DEXA is a light-sensitive compound, and, consequently, it forms variant transformation products (TPs) under light conditions. DellaGreca et al. [44] identified the photo abiotic factor-driven formation of TPs for DEXA following an 8 h exposure to irradiation using a solar simulator. Further, the formed TPs were observed to induce deleterious effects on *Ceriodaphnia dubia*. Elsewhere, other works have demonstrated that the formation of light-induced TPs and pathways for DEXA is dependent on the light source [48,58,59].

DEXA can be biodegraded by bacteria found in, e.g., activated sludge. In addition, the nature of the TPs formed is dependent on whether the bacteria are consortia or pure cultures. For instance, Pervaiz et al. [55] demonstrated the formation of variant TPs of DEXA, including dexamethasone-21-oic acid 17-oxodexamethasone, 6β-hydroxydexamethasone, and 16,17-unsaturated dexamethasone, under a consortium of bacteria (see Figure 1). However, pure cultures of *Bacillus subtilis* produced three major metabolites, namely, 6-hydroxydexamethasone, 17-oxodexamethasone, and 6-hydroxy-17-oxodexamethasone, distinctive from those formed by bacteria consortia.

Increasingly, studies have confirmed the presence of DEXA in variant environment matrices, including hospital effluents, industrial effluents, wastewater treatment plants influents and effluents, groundwater, seawater, sediments, soil, surface water, and drinking water across the globe (summarized in Table 2). The presence of DEXA in groundwater, river water, and, eventually, drinking water is attributed to the partial treatment of wastewater from WWTPs [27,60,61,62]. For example, DEXA has been observed to be inadequately removed in biological activated systems [62]. This is because WWTPs, by design, treat major organic pollutants in the mg/L range [27,60,63]. However, DEXA, its metabolites, and the likely formed TPs during treatment processes are in the ng/L–μg/L concentration range (Table 2), although highly stabilized; thus, they largely pass untreated.

For instance, DEXA has been detected in concentrations exceeding 3700 ng/L in hospital wastewater discharges [27], but most investigations have reported relatively low concentrations (Table S1) (<100 ng/L). Further, concerns over potential deleterious effects of DEXA over the longer term to both environmental health and potential risks to humans [30], for example, through drinking water and marine food, cannot be ruled out as, at present, they remain unquantified. Ismail et al. [30] identified DEXA and other emerging contaminants in the mariculture sediments of Pulau Kukup, Johor, Malaysia. In particular, DEXA in the sediments (0.144 ng/g) were largely considered to be from wastewater as well as human and animal waste discharges [30]. Elsewhere, DEXA in the range of 0.40–1.96 ng/L in Langat River, Malaysia, has also been reported [32]. Thus, these and other studies may suggest a likely wide prescription of DEXA as a corticosteroid anti-inflammatory drug in Malaysia [30,64,65].

DEXA and other endocrine-disrupting compounds (EDCs) have been identified in surface waters in Shanghai, China, at low concentrations (0.06–0.19 ng/L for DEXA) [33]. In the Netherlands, DEXA was detected in surface water (0.39−1.3 ng/L) and wastewater (11−243 ng/L), with large portions removed during wastewater treatment processes [66,67]. Similarly, these findings have been collaborated elsewhere [63,68]. Jha et al. [68] observed high concentrations of DEXA (2040–3770 ng/L) in hospital discharges in Lucknow, India; however, no DEXA was detected in river water samples collected at different points along the Gomti river, India. Elsewhere, in countries like Switzerland (147 ng/L) [69], Portugal (352 ng/L) [70], and Spain (360 ng/L) [28], however, much lower DEXA concentrations in hospital wastewater, compared to concentrations in India [68], have been observed. Taken together, based on these case studies findings, WWTPs may effectively remove DEXA, although the dilution effect in surface waters to levels below the detection limit(s) of the most widely used techniques cannot be ruled out.

The preceding paragraphs point to the increasing presence of DEXA across the world, from surface water to tap water (Table 2), as a result of the wide prescription of DEXA as a glucocorticoid to treat variant diseases (Table S1). However, lack of occurrence data in many regions (e.g., North and South Americas, with a single study in Africa) in addition to very few per matrix type studies (e.g., sediments)—with none in marine and estuarine system matrices—limits the possibility of drawing informative conclusions on the extent of DEXA distribution globally (Table 2). Overall, occurrence data raises the possibility of aquatic taxa exposure to DEXA, with largely unquantified risks and effects, especially at the sub-lethal level, to be discussed in Section 3.

Overall, despite the very low or negligible reported DEXA concentrations, continuous introduction of the parent drug and its associated metabolites may eventually result in pseudo-persistent behavior. This, in turn, can affect drinking water quality, with probable risk to human health, livestock, and wildlife over extended periods [51,57]. Additionally, wastewater from hospitals has been identified as having the highest concentrations of DEXA, implying that the increasing use of DEXA in response to the COVID-19 pandemic will, in turn, likely increase its concentration in different environmental matrices. This is particularly true given the increasingly high levels of COVID-19 daily infections, with a considerable number of patients requiring hospitalization and a sizeable portion meeting the prescription criteria for DEXA administration, as outlined by Horby et al. [9].

**Table 2:** Global concentrations of dexamethasone in environmental matrices.

Country	Environmental Matrix	Analytical Methods	Concentrations (ng/L)	References
China	Surface water	SPE-LC-MS/MS; UPLC MS/MS	<0.02–22	[71]
Malaysia	Surface water	ELISA kit	0.02–8.74	[65]
China	Urban river water	LC–MS/MS	0–8.0	[60]
China	Wastewater and river water	LC–ESI–MS/MS	0.3–3.4	[72]
Switzerland	Rivers and wastewaters	LC–MS/MS	8–13; 15–1720	[27]
The Netherlands	Various wastewater	LC–MS/MS	0–90	[67]
The Netherlands	Industry, hospital, STP, Surface water	CALUX reporter gene Bioassay	243, 96, 11–38, 0.39–1.3	[66]
China	River water	UHPLC–ESI–MS/MS	0.3–3.5	[61]
Germany	Treated wastewater, rivers, and streams.	LC–MS/MS	0–0.4	[73]
Singapore	Wastewater	UHPLC–(ESI)–MS/MS	236	[63]
Malaysia	Drinking water	ELISA kit	0–0.37	[65]
Malaysia	Drinking water	ELISA kit	0.01–0.26	[64]
Malaysia	Tap water	ELISA kit	0.32	[74]
South Africa	Effluent wastewater	LC-Orbitrap-HRMS	0.92	[75]
Malaysia	Drinking water	UHPLC–MS/MS	0.36–2.11	[76]
Hungary	River water	SPE–LC–MS/MS	0.064–0.068	[31]
France	Wastewater	SPE–LC–MS/MS	7–15	[77]
China	Sewage sludge	UHPLC–(ESI)–MS/MS	0.02–0.81	[78]
China	Surface water, Wastewater, and sludge	RRLC–MS/MS	0–22	[79]
China	Swine wastewater	RRLS–MS/MS	<1.27–260	[80]
China	Surface water (downstream—1000 m)	RRLS–MS/MS	37.8	[80]
Italy	River and wastewater effluent	SPE–HPLC–ESI–MS/MS	2–3	[81]
Japan	Wastewater effluent	UHPLC–MS/MS	0–1.3	[82]
Malaysia	River water	LC–QTRAP MS/MS	0–2.4	[83]
China	Aquaculture water	UPLC–MS/MS	0–41	[84]
Portugal	Hospital effluents, WWTP influent and effluent	UPLC–QTrap–MS/MS	0–352	[70]
China	Surface watersheds	UPLC–ESI–TQD–MS/MS	1.01–1.30	[85]
USA	Surface water, groundwater and wastewater	(SPE) UHPLC–MS/MS	<1–94	[76]
Spain	Hospital effluents	LC–(ESI)–MS/MS	360	[28]
Mexico	Hospital effluents	LC–MS/MS	9.8	[86]
Malaysia	Estuarine water	(SPE)-LC-MS/MS)	1.0–1.51	[29]
Spain	River waters, influent and effluent sewage	UHPLC–(ESI)–MS/MS^a^	<20	[51]

ELISA: enzyme-linked immunosorbent assay, ESI: electrospray ionization, HPLC: high-performance liquid chromatography, HRMS: high-resolution mass spectrometry, LC: liquid chromatography, MS: mass spectrometry, RRLC: rapid resolution liquid chromatography, SPE: solid-phase extraction, TQD–MS: triple quadrupole tandem mass spectrometry, and UHPLC: ultra-high-performance liquid chromatography, WWTP: wastewater treatment plant.

## 3. Ecotoxicological Effects of DEXA 

**Figure 2:**
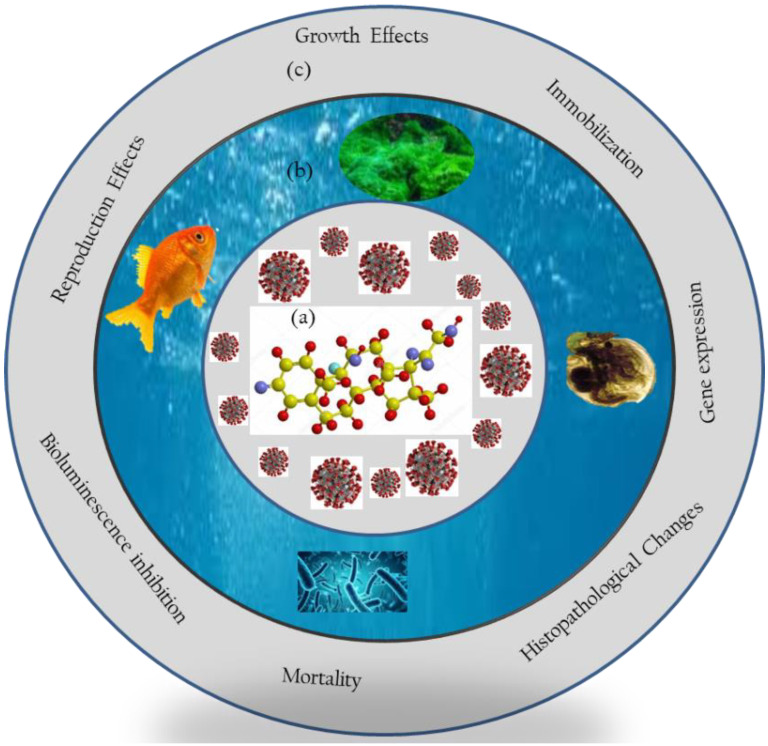
Schematic representation of (**a**) DEXA used for treating COVID-19, with likely interactions to (**b**) aquatic organisms at different levels of organizations, resulting in (**c**) the induction of diverse toxicological outcomes, including whole-body to molecular and cellular levels.

Studies have demonstrated the deleterious effects of DEXA on non-target aquatic taxa (e.g., fish, crustaceans, algae, and bacteria) at different levels of organization (Table S1). In summary, potential effects of DEXA to aquatic taxa include an increase in bacterial doubling time [42]; inhibition or promotion of growth and *chlorophyll-a* content in algae [44,48]; mortality, reproduction inhibition, and population growth in crustaceans [44,87]; and inhibition of growth and reproduction performance in fish [47], among others (Figure 2). Findings have demonstrated potential deleterious toxicological outcomes of DEXA to aquatic taxa, dependent on various factors. Herein, the illustrative deleterious toxicological outcomes of DEXA to aquatic taxa are briefly summarized.

### 3.1. Effects on Bacteria and Microbial Communities

Currently, only a handful of studies have examined the potential effects of DEXA on microorganisms and microbial communities [42,43,88]. Plotkin et al. [42] tested the effects of DEXA at 0.785, 7.85, and 19.62 mg L^−1^ on the growth rate and antibiotic susceptibility of *Enterococcus faecalis*, *Escherichia coli*, *Pseudomonas aeurginosa*, and *Staphylococcus aureus* in Mueller-Hinton broth. Results revealed that the doubling times of *E. faecalis* and *P. aeruginosa* were significantly increased at all concentrations. However, for S. *aureus,* effects were observed at the highest concentration, and there were no effects on *E. coli,* irrespective of the exposure concentrations. The effects were linked to likely changes of cellular membrane properties by the hormones used.

Effects of DEXA before and after photodegradation were investigated on a luminescent bacterium, *Photobacterium phosphorium,* with none observed under non-illuminated conditions [43]. However, an EC_50_ of 133.8 mg L^−1^ (tested range of 7–455 mg/L) was obtained following DEXA photo-illumination. Observed effects were associated with the formation of photo-induced TPs; however, the TPs were unspecified but were potentially considered more toxic than DEXA. Although these effects were observed at concentrations exceedingly above the MECs for DEXA in actual environmental matrices of >100 ng/L (Table 2), the potential implications, due to an increased environmental presence and the transformation of DEXA to microbial communities, remain of concern. 

Li et al. [88] examined the effects of 500 ng L^−1^ DEXA on microbial communities and its removal in three water river samples, all in China, over 50 d. Findings demonstrated that DEXA decreased significantly or completely in all water samples compared to the controls (without microorganisms). Observed differences in the degradation of DEXA were dependent on microbial community abundance, composition, and water physicochemical characteristics, e.g., pH and total organic carbon [88]. Minimal effects of DEXA on bacteria were linked to its hydrophilicity and reduction in the media due to adsorption by organic colloids. Proteobacteria, common in aquatic and sediment environments, were found to thrive in all aquifer media, likely due to their organic compound degrading abilities. The abundance of other genera, e.g., *Methylophilus,* was also shown to be more pronounced due to their ability to degrade DEXA.

Overall, these results show that the microbes prevalent in water, e.g., Proteobacteria, may reduce the levels of DEXA. However, there is an urgent necessity to examine the long-term effects of DEXA on other microorganisms. This is essential since degradation studies, e.g., by Kawabata et al. [43], have indicated TPs from DEXA may be highly toxic to aquatic taxa, especially under solar irradiation conditions. Secondly, there is a need to consider DEXA effects in the environment following co-existence with other pollutants as, in turn, this may result in attenuated or potentiated toxicological outcomes to microbial communities. 

### 3.2. Effects on Algae

The effects of DEXA before and after gamma irradiation—known to degrade stable organic pollutants in aqueous media—were investigated on two algal species [48]. *Microcystis flos-aquae* and *Scenedesmus obliquus* were exposed to high concentrations (25, 50, and 100 mg/L) of non-irradiated DEXA and 50 mg/L of irradiated DEXA solution at 4000 gamma in BG 11 algal medium over 14 d. The results demonstrated DEXA concentration-dependent algal growth promotion and an increase in *chlorophyll-a* content in both algal species for non-irradiated solutions. However, the effects of irradiated DEXA were species-dependent. Chlorophyll-a content of *M. flos-aquae* increased by 4.27%, whereas *S. obliquus* yielded a decrease of 25.65%, with the latter being more sensitive to irradiated DEXA compared to the former species [48]. Remarkably, growth promotion and inhibition of *M. flos-aquae* and *S. obliquus*, respectively, may both lead to undesirable ecological imbalances. For instance, as *M. flos-aquae* is the main algae in eutrophic water, excessive growth may result in altered species composition and overall undesirable implications to ecological functioning [89]. Conversely, the extinction of *S. obliquus* may lead to undesirable population alterations of consumers (e.g., *Daphnia magna*) and higher organisms (e.g., fish) in the food chain, triggered by food scarcity.

DellaGreca et al. [44] investigated the behavior and growth inhibition of DEXA and its photoderivatives in *Pseudokircheneriella subcapitata* over 72 h. DEXA suspended in water was first irradiated by a solar simulator for 8 h and achieved 15% conversion, where two photoderivatives (TP 11 and TP 12) were isolated. Results revealed DEXA induced no observable effects on algal growth (EC_50_ > 100 mg/L), but TPs 11 and 12 had much lower EC_50s_ of 12.15 and 40.75 mg/L, respectively. These findings illustrate that non-toxic DEXA may undergo transformation processes in ecosystems and, in turn, form TPs that are highly toxic to aquatic taxa. 

### 3.3. Effects on Aquatic Invertebrates 

Three endpoints, including the acute effects, immobility, and mortality of DEXA and associated TPs (TP 11 and TP 12), were investigated on rotifer, *Brachionus calyciflorus*, and two crustaceans, *Thamnocephalus platyurus* and *Daphnia magna*, over 24 h. Chronic tests on the inhibition of reproduction and population growth endpoints were assessed for a crustacean, *Ceriodaphnia dubia*, over 7 d [44]. Results revealed that the 24 h-EC_50_ results for *D. magna* were 48.30, 10.88, and 17.82 mg/L, respectively, for DEXA, TP 11, and TP 12. Thus, the TPs induced higher toxicity than the parent DEXA drug under acute testing conditions (Table S1) (24 h), irrespective of the target organism [44]. 

Investigations on LC_50_ showed DEXA, TP 11, and TP 12 were toxic to *T. platyurus* (60.11, 20.9, and 30.52 mg/L, correspondingly) and *B. calyciflorus* (48.22, 13.20, and 44.66 mg/L, respectively). However, the 7 d-EC_50_ results for DEXA, TP 11, and TP 12 were highly toxic (0.05, 0.13, and 0.06 mg/L, respectively) against *C. dubia*. Acute test findings indicated TPs induced higher toxicity compared to the parental DEXA compound. Conversely, under chronic testing conditions, DEXA either exhibited toxicity in the same order or higher compared to TPs [44]. These findings underpin the essence to assess both the parent compound and associated TPs under chronic exposure conditions to systematically elucidate their potential implications or lack thereof to ecosystems. Further, *C. dubia* was more sensitive compared to algae, irrespective of the parent drug or formed TPs. Thus, inhibition of *C. dubia* growth may result in the deleterious disruption of the food chain as it forms a link between primary producers (e.g., algae) and aquatic vertebrates (e.g., fish) [90]. 

Two parallel sets of toxicity bioassays were run for up to four consecutive generations (F0–F3) of *C. dubia*. Short- and long-term effects of DEXA to crustacean *C. dubia* included acute immobilization (50–3200 µg/L) and multigenerational chronic effects (fecundity, average time to first brood, mean brood size, body length) (1.95–125 µg/L) [87]. Based on the 48 h-EC_50_ results, DEXA was very toxic (EC_50_ = 750 µg/L), but no significant differences were observed in the controls as a function of generation. A significant decrease in life-history parameters (e.g., fecundity, the average time to first brood) was observed to be dependent on DEXA concentration and exposure duration. *C. dubia* showed excellent survival at tested concentrations in generation F0–F3, except at the end of the F1 generation at 125 µg/L. Reproduction inhibition was observed in F0 at a test concentration of 62.5 µg/L. Further, due to high reproduction inhibition and consequent mortality at 125 µg/L in F1, progeny from the third brood (F2) was inadequate to continue the next generation (F3) [87]. 

DEXA exposure to F3 yielded mean offspring numbers of 4.6 and 9.2 at 62.5 and 15.6 µg/L, respectively, compared to 17.8 in control groups, with a significant reduction in reproduction at 3.9 µg/L (*p* < 0.05) (Bal et al., 2017). The higher toxicity of DEXA at lower concentration(s) is likely due to the presence of the fluorine atom at the 9α position, responsible for increased glucocorticoid, which, in turn, slows down metabolic activity [91]. Changes in the reproductive behavior of the crustaceans’ parent generation have been reported to be accompanied by a low quality of progeny and generally low population fitness [92]. Thus, the observed reduction in reproduction ability may cause population extinction and undesirable ecosystem imbalance, likely further exacerbating the planetary challenges linked to dramatically deteriorating biodiversity loss.

### 3.4. Effects on Fish

Until now, highly variant deleterious toxicological outcomes of DEXA to fish from physiological [47,93,94,95] and morphological [45] to molecular [46] levels have been observed, including under mixture scenarios with other pollutants, as summarized in Table S1. On the organismal scale, for instance, DEXA has been found to induce adverse effects on the growth and reproduction performance of seabream fish [47]. DEXA at 800 mol/m^3^ over 35 d exposure caused higher energy expenditure, resulting in lower growth rates. Moreover, DEXA- and cortisol-fed fish had stimulations on their amino acid catabolism and gluconeogenic pathways, both in muscles and gills, but with markedly enhanced effects on DEXA fed to fish. This suggests that under chronic stress conditions, variant corticoids exert diverse effects on fish, with DEXA being the most toxic.

Although DEXA is released at low concentrations to ecosystems (ng/L–µg/L), this can, however, potentially alter fish populations. Guiloski et al. [96] demonstrated a reduction in testosterone levels with the resultant impairment of reproduction in adult *Hoplias malabaricus* following exposure to DEXA (at 0.03, 0.3, and 3 µg/kg). DEXA can also potentially interfere with embryonic development, resulting in increased deformities in fish, as recently observed in the *fathead minnow* species [45]. Milla et al. [97] observed that elevated concentrations of cortisol can decrease 11-ketotestosterone production, which results in smaller gonads and poor sperm quality in teleost fish production. In actual ecosystems, this may mean a marked reduction or failure in reproduction, with serious implications not only on the individual organism’s population but likely adverse impacts, including imbalances, at the community level.

The effects of DEXA and its variant mixture ratios with other pollutants on the metabolism and enzymatic activities of different fish species have been reported [96,98]. For example, a mixture of DEXA (0.039 mg/L) and indole-3-carbinol (19.62 mg/L) over 10 min was observed to induce a reduction in EROD activity. The findings indicated the potentially harmful effects of DEXA on Cytochrome P450 (CYP) and the detoxification of fish (Rainbow trout) [98] (Sakalli et al., 2018). Guiloski et al. [96] observed an elevated antioxidant system in fish livers due to oxidative stress induced by DEXA, including catalase (CAT), glutathione peroxidase (GP_x_), glutathione S-transferase (GST), glutathione (GSH), and lipoperoxidation (LPO). However, the antioxidant systems (GP_x_, GST, and GSH) were reduced in the gonads. Overall, the induced oxidative stress and metabolism changes were found to be organ-dependent and highly variant following exposure to DEXA. Other works have revealed that antioxidant SOD, GP_x_, and GST decreased in the heart following exposure to DEXA but increased significantly in the kidney [99]. Thus, DEXA can cause oxidative stress with adverse implications to fish, and the effects are organ-dependent.

Carbohydrate and amino metabolisms were higher in DEXA-fed seabream (*Sparus aurata* L.) fish, resulting in hepatic glycogen and glycogen phosphorylase being higher in both the liver and the muscles [47]. The work of Das et al. [100] reported liver hypertrophy from enhanced glycogen turnover in hepatocytes in DEXA-fed gilthead seabream. Concerning amino acid metabolism, fish exposed to DEXA resulted in modified branchial enzymes, with resultant enhanced lactate, amino acid, and free fatty acid metabolism [47]. Additionally, the administration of DEXA for 35 d stimulated amino acid catabolism in the muscles of the gilthead seabream. Taken together, this implies that widespread contamination of ecological systems by DEXA may impair fish metabolic mechanisms.

Several investigations have examined molecular and anti-inflammatory level effects of DEXA on variant fish species that have resulted in the modification of certain genes and proteins. Zebrafish larvae and adult male zebrafish, following exposure to DEXA, expressed distinctively different genes specific to the species type, dependent on developmental and tissue organ specificity (Table S1) [46]. Results revealed that the expression of *pepck*, *baiap2,* and *pxr* were changed in zebrafish larvae, whereas *baiap2, pxr,* and *mmp*-*2* were perturbed in the male adult zebrafish liver following exposure to 5, 50, and 500 mg/L over 4 d [46]. The anti-inflammatory molecule results demonstrated that the immune gene expression of *il*-1β, *cxcl*-*8*, *tnfα,* and *crp* was downregulated following DEXA injection on crucian carp (*Carassius auratus*) [101]. A study by Kumari et al. [102] examined the levels of melatonin-related genes (*MT 1*, *2*, *aanat 1,* and *aanat 2*) and the stress-related (*nfk*b) gene after exposure of adult zebrafish to DEXA at early life and then treatment with *Piper betle L*. (PB). DEXA-treated adult zebrafish exhibited anxious behavior, lower melatonin, and higher mRNA expression of the *nkfb* gene compared to the control. Further, results showed increased *MT 2* expression during exposure and the suppression of *aanat 2*; however, no changes in *MT 1* and *aanat 1* expressions were observed [102]. Hence, exposure to DEXA in the environment may perturb the expression of genes in, e.g., anti-inflammatory, immunity, and molecular aspects in fish. Hence, these endpoints can serve as potential sensitive biomarkers for DEXA biomonitoring.

## 4. Treatment Technologies of DEXA in Technical Systems

Here, technologies employed to treat DEXA, its metabolites, and TPs in WWTPs from lab-scale to full-scale plants are summarized. DEXA is broadly treated using chemical, physical, and biological technologies [103,104,105], with highly variant removal efficacies of up to 100% both in synthetic and actual wastewater matrices [52,58,69,78,79,106] (Table S1). Generally, DEXA is likely to be persistent in the environment, essentially enhanced by its molecular structure—the fluoro-functional group that stabilizes the aromatic ring, thus resisting degradation. As such, their low concentrations in the environment, in both technical and natural systems, is due to low usage doses of DEXA (Table 2); it is not degraded because it has a fluoro-molecular structure, as is the case for pollutants with a similar structure such as ciprofloxacin. 

### 4.1. Conventional Removal of DEXA in Wastewater

Primary treatment processes include flocculation and coagulation (e.g., to screen and settle out solid wastes, floatables) to prevent the inhibition of downstream processes. Conversely, secondary treatment processes chiefly entail microbial-based systems designed to remove microbial organisms, break down organic contaminants, as well as nitrogen and phosphorous nutrients. However, these processes have low removal efficiencies for pharmaceuticals and other micropollutants and are highly dependent on the chemical nature of the pharmaceutical compound in question. Due to the low octanol–water partitioning coefficient (log K_ow_ ≈ 1.9) of DEXA, it has a low sorption potential [107,108]. This means minimal or no removal of DEXA through sorption or partition occurs at the primary treatment stage [104], according to data summarized in Table S2.

Secondary treatment configurations follow primary treatment and include activated sludge bioreactors, rock bed trickling filters, membrane biological reactors (MBR), sequencing batch reactors, constructed wetlands, ponds, and lagoons [78,79]. Results show that secondary treatment systems exhibit highly variant removal efficiencies of DEXA, depending on the specific technology employed (Table S2). Bain et al. [104] evaluated the removal of hormones, including DEXA, at different treatment stages in three Australian wastewater treatment plants, defined by marked differences in configurations. 

The first plant was fitted with a sequencing batch reactor and reduced DEXA to below the detection limit (initial concentration was 60 ng/L). However, following the chlorination process, DEXA was detected in the effluent, plausibly due to deconjugation of its metabolites and biotransformation products—both known to react with chlorine to reconstitute the parent compound [109]—with similar findings observed elsewhere [110]. The second plant consisted of rock bed trickling filters, humas tanks, maturation ponds, and constructed wetlands, with 100% removal of DEXA achieved beyond the maturation ponds. Other works have applied lagoons to treat DEXA in livestock wastewater. Liu and colleagues [80], using lagoons to treat DEXA in flush water generated from a swine farm at a concentration of 260 ng/L, observed 85% removal efficiency, with 37.8 ng/L detected in the receiving environment at 1000 m from the source.

In the larger plant, Bain et al. [104] recorded the minimal removal of glucocorticoids, including DEXA, with a daily capacity of 35 megalitres (serving a population of 160,000). Here, influent concentrations ranging from 51 to 85 ng/L DEXA equivalents were recorded, with about 70 ng/L detected in the effluent. Markedly, these findings showed limited removal, although the influent was treated through aerobic bioreactors and biological nutrient reduction before the chlorination stage. Other works have evaluated the removal of DEXA in activated sludge in WWTPs comprising anaerobic, anoxic, and aerobic treatment stages [103]. Kenaga and Goring [111] investigated a WWTP influent with an average glucocorticoid concentration of 42 ng/L, with 0.81 ng/L attributed to DEXA. Ninety-five percent DEXA removal was observed in the first two treatment stages (anaerobic and anoxic). Similarly, Halfon et al. [112] demonstrated that a treatment plant yielded 90% DEXA removal (from 22.6 to 2.3 ng/L) after the first two stages. Notably, no DEXA was detected in the final effluent in both studies as, in each case, the effluent was not chlorinated before discharge. Hence, there was no possibility for the reformation of DEXA. Additionally, the results on sludge analysis for DEXA indicated none was adsorbed into the solids, an indication that biotransformation was the likely dominant removal mechanism.

### 4.2. Advanced Water Treatment Techniques 

Current literature, as discussed in Section 4.1, shows conventional WWTPs can efficiently remove DEXA from municipal influent, especially at low concentrations [113,114]. However, influents with higher DEXA concentrations, e.g., from hospitals and the animal husbandry sector (e.g., swine farms), are ineffectively remediated using biological secondary treatment processes, with concomitant significant environment concentrations detected in the effluent [87]. Depending on the water quality requirements for the effluent discharges with respect to public and environmental health, tertiary treatment technologies are required to remove the remaining DEXA post-primary processes [24,103,115], as the results in Table S2 demonstrates. The results reveal highly variant successes in the treatment of DEXA in synthetic wastewater and WWTP influent, depending on system size from batch to pilot plants and actual large-scale WWTPs. Herein, key findings on DEXA removal using advanced technologies, including membrane technology [78,79], physicochemical treatment [78,79], and advanced oxidation processes [24,80,109], are summarized. 

#### 4.2.1. Physico-Chemical Treatments

Activated carbon adsorption is commonly employed to control taste and odor in drinking water and to treat secondary effluent, especially in water reuse applications. Researchers have observed that granular activated carbon (GAC) and powdered activated carbon (PAC) can effectively remove numerous pharmaceuticals, including DEXA at trace concentration ranges [116,117]. GAC removed DEXA sodium phosphate from very low to high concentrations (0.01–100 mg/L). Findings showed complete removal at lower concentrations but with only 35.7% efficiency achieved at 100 mg/L [103]. Other works evaluated activated carbon (AC) and carbon nanotube (CNT) technologies on the removal of DEXA [69]. The results indicated an adsorption capacity of 4 and 1.8 mg/g for AC and CNTs, respectively [50]. Notably, while AC appeared as an attractive option to treat DEXA, high organic matter in wastewater may potentially limit its performance owing to the competition for sorption sites and the blockage of pores within the sorbent structure [118]. 

Natural and modified clays (e.g., zeolite and montmorillonite) have been investigated for DEXA removal from aqueous solutions, with 63% to 100% effectiveness achieved with initial dose concentrations between 50 and 100 mg/L [48,50,106,119]. For example, 50 mg/L of DEXA in synthetic water were removed using naturally occurring montmorillonite, yielding an efficacy of 90.6% [50]. This suggests that clay may be effective to remediate low DEXA concentrations found in the environment. Overall, clays are a viable alternative, unlike AC, as the former is cheaper, besides being easy and safe to dispose of [113]. Remarkably, the conventional chemical coagulation–flocculation process is known to remove a wide range of pharmaceuticals during drinking water treatment [50] but is ineffective for DEXA removal in aqueous solutions. However, modifying this process (electrocoagulation) revealed variant treatments of DEXA were effluent-type-dependent (e.g., synthetic effluents, hospital effluents). For example, using electrocoagulation, Vadi et al. [120] observed 86% removal of DEXA from a synthetic solution (1400 mg/L), whereas treatment of hospital wastewater fortified with 0.1 mg/L DEXA achieved 38% efficacy [120].

#### 4.2.2. Membrane Technology

Membrane removal of micropollutants, including DEXA from aqueous systems, is hinged on a single or a combination of variant mechanisms, including size exclusion, adsorption onto the membrane, and charge repulsion [24]. Sulaiman et al. [50] observed high DEXA rejection using both nanofiltration (NF) and reverse osmosis (RO) membranes, irrespective of the water matrix in focus. This was attributed to DEXA’s large molecular size compared to the tight structure of NF and RO membranes, as well as high dipole moments and relatively low water solubility [118]. 

Similarly, Mohseni et al. [121] demonstrated that combined microfiltration (MF) and RO membranes yielded a reduction in DEXA concentration close to three-fold (0.16 ng/L to <0.06 ng/L). MF is inefficient in removing DEXA since the membrane pore sizes are larger than the target compound [109,118]. Hence, it is a common practice to place MF ahead of RO as a pre-treatment process to eliminate potential foulants, e.g., suspended solids. On the downside, although the resultant permeates from membrane-based treatment methods have had minimal DEXA concentrations, the formed brine is a secondary waste stream requiring further treatment due to elevated DEXA concentrations. This renders membrane use prohibitive and less cost-effective, similar to findings for other APIs [52,122,123].

Membrane biological reactors (MBRs) are hybrids of biological activated sludge wastewater treatment coupled with MF or NF membrane separation processes. These systems are presently popular for the removal of micropollutants [117,124] due to lower space requirements, and they produce higher quality effluent compared to conventional plants [117]. MBRs have a long sludge retention time (SRT), which, in turn, aids the highly diverse consortia of microorganisms (including slow-growing bacteria taxa) essential for effective treatment [103]. Luo et al. [110], using a membrane bioreactor, reported 100% removal of DEXA from hospital effluent with an initial concentration of 147 ng/L. Under COVID-19, anticipated increases in the use of DEXA for treatment may lead to elevated influent concentrations, with treatment systems incorporating MBR technology plausibly effectively treating hospital effluent before it is discharged into municipal sewage systems.

#### 4.2.3. Advanced Oxidation Processes 

**Figure 3:**
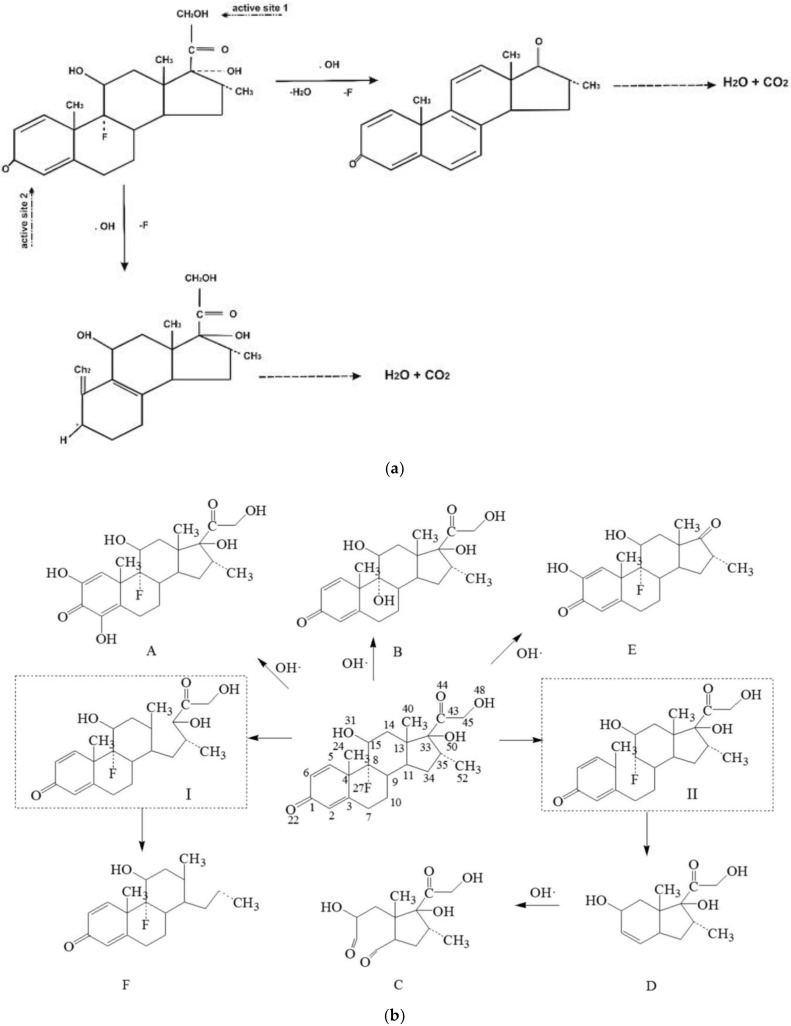
Degradation pathways of DEXA using advanced oxidation processes: (**a**) photocatalysis system using nTiO_2_ [132], and (**b**) gamma irradiation [48].

Advanced oxidation processes (AOPs) are among widely researched technologies for the removal of micropollutants [103] due to their capability to degrade recalcitrant compounds, e.g., micropollutants [113,125]. AOP technology relies on the generation of highly oxidizing free radicals (e.g., •OH), which initiate a non-selective redox reaction with the target pollutant. To remove DEXA, different AOP technologies have been applied [126,127,128]. For example, Besha et al. [126] demonstrated that photocatalysis using titanium dioxide nanoparticles (nTiO_2_) irradiated with UV light completely degraded 10 mg/L of DEXA; illustrative possible reaction pathways are shown in Figure 3a. Alternative catalysts, e.g., nanoparticles of zirconium dioxide (nZrO_2_) and tungsten trioxide (nWO_3_), are equally effective under similar irradiation conditions [129]. Though effective, UV-initiated photocatalysis is economically unfeasible due to the high energy requirement for treating large influent volumes. Hence, visible-light-initiated photocatalysis is thus a possible solution. Verlicchi et al. [109] reported 30% to 70% DEXA removal with an initial concentration of 15 mg/L (although an unlikely dosage in effluents) using nTiO_2_ doped with Ag under visible light irradiation.

Other AOP technologies have been investigated for DEXA removal in aqueous media. Several researchers have demonstrated variant removal efficiencies for DEXA that are dependent on ozone (O_3_) concentration [103,114,116]. The degradation of organic compounds using UV or gamma irradiation (photolysis)—another form of AOP [48,130,131]—has previously been employed to treat DEXA [116]. For example, Guo et al. [48] demonstrated the use of gamma radiation for the complete removal of low concentrations of DEXA through a set of pathways, as shown in Figure 3b. Jia et al. [103] observed a 95% removal of DEXA, even at high concentrations of up to 50 mg/L, using gamma irradiation. Chiefly among the merits of using AOPs is the mineralization of the target compound without generating a secondary waste stream. However, AOPs tend to form numerous intermediate products that are potentially more toxic than the parent compound [58,119]. As a result, this limits the technology in actual systems as numerous TPs’ toxicity of DEXA to aquatic taxa remains unquantified.

## 5. Environmental Risks of Detected DEXA

Does DEXA, due to current uses and environmental concentrations and concomitant with increasing use in response to COVID-19, pose existential potential ecological risks? Following conventional environmental risk assessment (ERA) techniques, the potential risk of DEXA was evaluated in surface waters. This was achieved by comparing the detected concentrations (measured environmental concentration (MEC)) to the predicted no-effect concentration (PNEC) values to determine the risk quotient (RQ). At present, the highest MEC for DEXA is 37.8 ng/L [80], with many regions having concentrations >1 ng/L in surface waters globally (Table 2). From the reviewed studies, the least ecotoxicity of DEXA to daphnia and fish were 0.05 mg/L (EC_50_) [44] and 0.1 µg/L No Observed Effect Concentration (NOEC) [133], respectively (Table S1). Thus, the resultant PNECs for daphnia and fish are 50 and 1 ng/L (based on E(L)_50_ and NOEC data in Table S1), respectively. Based on these data, the highest probable RQs for DEXA to daphnia and fish, respectively, are 0.76 and 37.8.

To contextualize the RQ results, ranking, following the formalism, low (RQ < 0.1), medium (0.1 ≤ RQ < 1), and high (RQ ≥ 1) risks [134,135] can offer insights on DEXA potential risks to aquatic organisms at different levels of organization. At the present MEC levels, RQs suggest DEXA poses medium (daphnia) to high (fish) level ecotoxicological risks, depending on the taxa in question in a given receiving surface waters. Markedly, effects on fish are highly elevated across many regions around the globe, with most MECs exceeding 1 ng/L (Table 1). Additionally, no apparent risks to wastewater were evident although DEXA has been detected at very high concentrations, e.g., >3.7 µg/L in hospital wastewater [68], as it is not harmful to bacteria (EC_50_ = 133.80 mg/L) [43]. 

Two key aspects require careful consideration in concluding the probable risk levels of DEXA to aquatic taxa. Due to the dearth of acute and chronic toxicity data for DEXA to numerous taxa in wastewater, soils, estuaries, and marine environmental matrices, definitive evidence on its potential adverse implications to organisms dwelling in these habitats is still lacking. Moreover, owing to limited studies on the toxicity effects of DEXA in actual environmental matrices, e.g., actual freshwater systems, together with the use of highly variant exposure protocols (Table S1), potential deleterious implications cannot be ruled out. The reason is that exposure media, the exposure experimental approach, and the age of the organisms, among other factors, significantly exert influence on the likely or absence thereof of pollutant effects to the given taxa in focus. Additionally, an aspect not accounted for in conventional ERA studies [136,137] are the sub-lethal effects, as only whole-organism level lethality effects are incorporated in these models.

Hence, sub-lethal effects triggered by DEXA at molecular to cellular levels remain underestimated [136]. This has far-reaching implications as the reviewed studies herein suggest that DEXA can induce deleterious sub-lethal effects such as modulating endocrine system pathways in fish. Additionally, effects may occur over a lifetime and across multiple generations, unlike the static considerations reflected in the ERA models. This implies DEXA can interfere with reproductive success, resulting in poor outcomes across multiple generations, with far-reaching deleterious implications of population-level effects [133]. Overall, based on ERA and sub-lethal effect assessments, it appears that with the increasing use of DEXA in response to COVID-19, in addition to routine uses, the resultant higher concentrations in surface waters may potentially trigger elevated risks to aquatic taxa, including those that are poorly investigated, e.g., algae, one of key primary food source in the aquatic systems widely known to be highly sensitive to organic pollutants, including different classes of APIs.

## 6. Concluding Remarks and Future Perspectives 

The data reviewed herein demonstrate the occurrence of DEXA in different environmental matrices (e.g., sediments, surface water) and drinking water at variant concentrations, from <1 ng/L up to >3.7 µg/L. Remarkably, occurrence data for DEXA are too few even in regions where it has been reported (e.g., Europe and Asia), and this is expected to increase significantly in response to COVID-19. This necessitates further studies to determine the extent of DEXA pollution, especially in compartments such as soils and estuarine and marine environments, where no data is available. Moreover, studies should be done globally as the use of DEXA varies markedly across regions, and data published elsewhere globally are not reflective of the consumption underpinned by disease variability and agricultural practices besides the economies’ intra-country variability. The USA, with the highest number of COVID-19 cases, increasingly high hospitalization (with >100,000 per day during December 2020–January 2021), and a large economy, has no occurrence data of DEXA to date for current uses.

Ecotoxicological effects from the literature are highly variant over several folds, e.g., intraspecies (from µg/L to mg/L) and among different species (fish > daphnia > algae). Hence, this reflects high sensitivities to DEXA depending on species biology, age, growth phase, methods of exposure used, and endpoints measured. As the MEC of DEXA is likely to exceed >1 ng/ L with increasing use, and where, even at such low concentrations, deleterious effects are likely to occur, this should motivate further works on the long-term effects on the organisms. Such studies can be at the physiological (e.g., fertilization, sperms production, reproduction), molecular (oxidative strengths, energy balance, mechanisms of toxicity using omics tools), and cellular (e.g., effects on mitochondria and some protein expressions) levels. To this end, the use of realistic environmental concentrations in experiments to accentuate the actual effects of DEXA, its metabolites, and TPs on aquatic taxa should inform future experimental designs. Until now, however, only a handful of studies have investigated the potential short- and long-term implications of parent DEXA, their metabolites, and TPs to aquatic taxa, both as individual pollutants and as a mixture(s). This is also true for the mixture effects of DEXA with other pollutants and not for the parent drug, its metabolites, and TPs, where synergistic effects cannot be ruled out. Thus, the exclusion of TPs and metabolites on the risk assessment of DEXA only offer a half-set of facts on its actual implications to entire ecosystems.

Additionally, results have shown that DEXA can be largely removed in WWTPs, especially in certain processes, to levels below detection limits. However, other systems can only reduce it to low levels, which are directly discharged into the surface waters. Remarkably, even in systems where DEXA has been reduced to below detection levels, some works have confirmed that the post-chlorination process causes its re-emergence in the effluent following the deconjugation of DEXA’s metabolites and biotransformation products. At present, this raises the need to ascertain current WWTPs’ technology effectiveness to treat DEXA, its metabolites, and TPs, as the available data are insufficient to allow us to draw affirmative conclusions. 

## Figures and Tables

**Table 1:** Summary of widely used or under trial phase potential candidate drugs for the treatment of COVID-19 patients across the globe.

Drug Category	CAS No	Drug Name	Formula	Molecular Weight (g/Mol)	Molecular Structure
**Antivirals**	1809249-37-3	Remdesivir	C_27_H_35_N_6_O_8_P	602.6	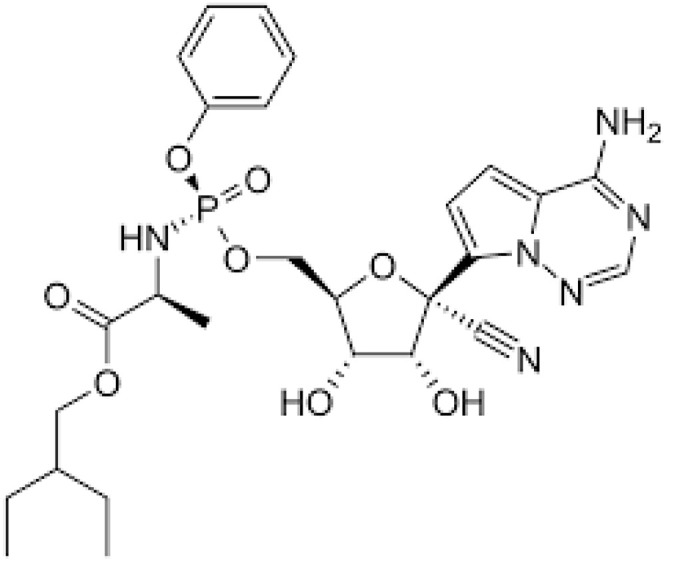
	159989-64-7	Nelfinavir	C_32_H_45_N_3_O_4_S	663.9	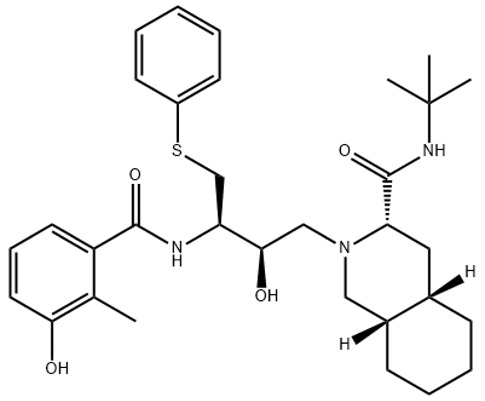
	259793-96-9	Favipiravir	C_5_H_4_FN_3_O_2_	157.104	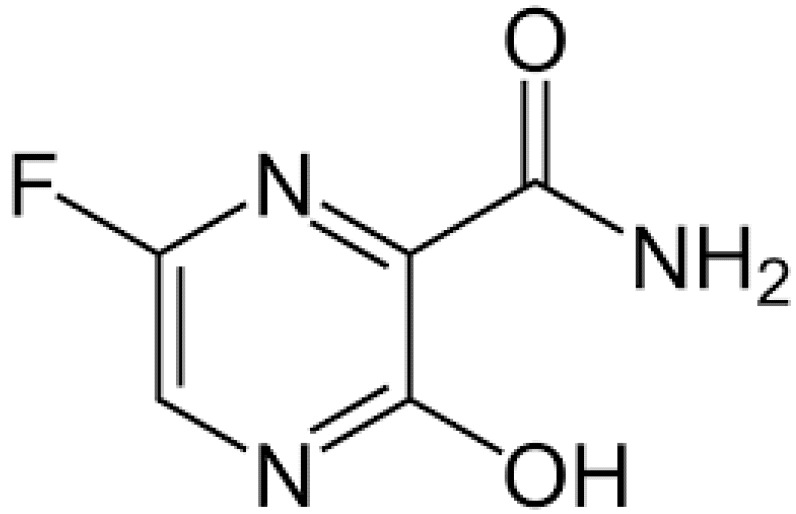
	192725-17-0	Lopinavir-	C_37_H_48_N_4_O_5_	628.814	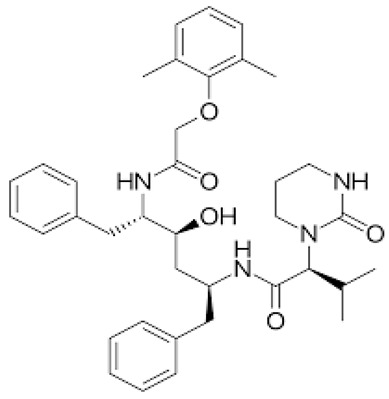
	155213-67-5	Ritonavir	C_37_H_48_N_6_O_5_S_2_	720.948	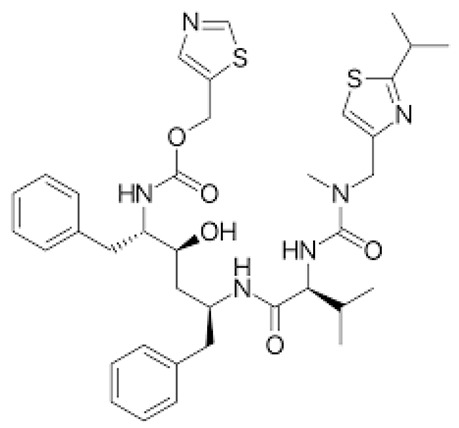
	196618-13-0	Oseltamivir	C_16_H_28_N_2_O_4_	312.4	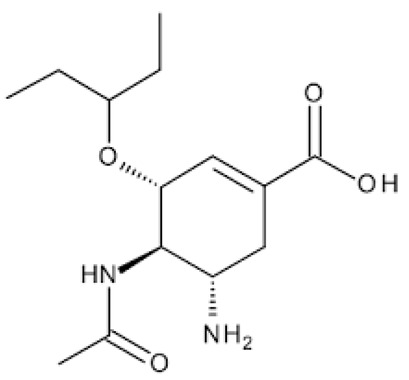
**Antimalarial**	54-05-7	Chloroquine	C_18_H_26_ClN_3_	319.872	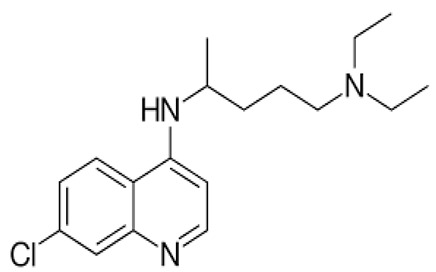
	118-42-3	Hydroxycloroquine	C_18_H_26_ClN_3_O	335.872	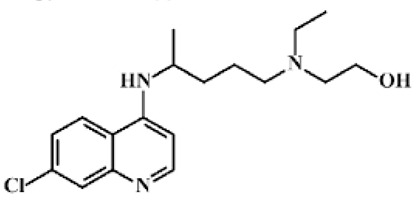
**Anti-Inflammatory**	50-23-7	Hydrocortisone	C_21_H_30_O_5_	362.46	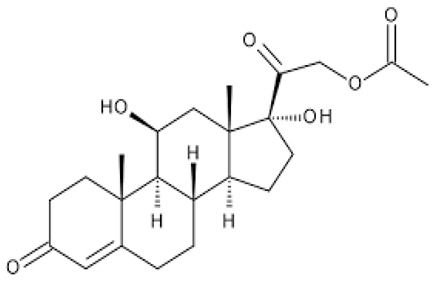
	50-02-2	Dexamethasone	C_22_H_29_FO_5_	392.46	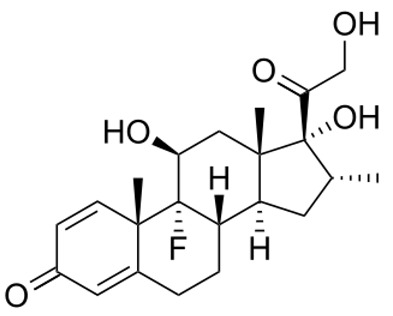
**Antiparasitic**	70288-86-7	Ivermectin	C_48_H_74_O_14_	875.1	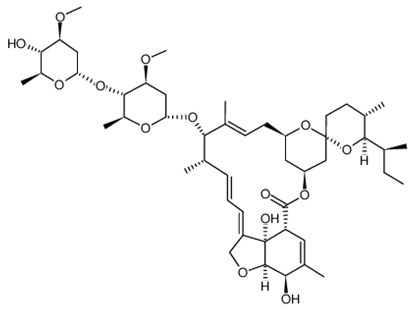
**Antibacterial**		Azithromycin	C_38_H_72_N_2_O_12_	749.0	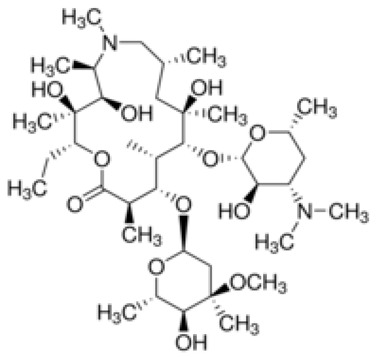

## Data Availability

Not applicable.

## Supplementary Materials

The following are available online at https://www.mdpi.com/article/10.3390/ijerph182111291/s1, Table S1: The ecotoxicological effects of DEXA on organisms at different levels of organization, Table S2: Removal of DEXA during wastewater treatment in WWTPs, with techniques utilized across geographical regions globally in selected studies.

## References

[1] Guan W., Ni Z., Hu Y., Liang W., Ou C., He J., Liu L., Shan H., Lei C., Hui D.S.C. (2020). Clinical Characteristics of Coronavirus Disease 2019 in China. N. Engl. J. Med..

[2] Zheng Y., Xiong C., Liu Y., Qian X., Tang Y., Liu L., Leung E.L.-H., Wang M. (2020). Epidemiological and Clinical Characteristics Analysis of COVID-19 in the Surrounding Areas of Wuhan, Hubei Province in 2020. Pharmacol. Res..

[3] Zhu N., Zhang D., Wang W., Li X., Yang B., Song J., Zhao X., Huang B., Shi W., Lu R. (2020). A Novel Coronavirus from Patients with Pneumonia in China, 2019. N. Engl. J. Med..

[4] Rodriguez-Morales A.J., Bonilla-Aldana D.K., Balbin-Ramon G.J., Rabaan A.A., Sah R., Paniz-Mondolfi A., Pagliano P., Esposito S. (2020). History Is Repeating Itself: Probable Zoonotic Spillover as the Cause of the 2019 Novel Coronavirus Epidemic. Infez Med..

[5] WHO Director-General’s Opening Remarks at the Media Briefing on COVID-19—11 March 2020. https://www.who.int/director-general/speeches/detail/who-director-general-s-opening-remarks-at-the-media-briefing-on-covid-19---11-march-2020.

[6] Dong E., Du H., Gardner L. (2020). An Interactive Web-Based Dashboard to Track COVID-19 in Real Time. Lancet Infect. Dis..

[7] Ksiazek T.G., Erdman D., Goldsmith C.S., Zaki S.R., Peret T., Emery S., Tong S., Urbani C., Comer J.A., Lim W. (2003). A Novel Coronavirus Associated with Severe Acute Respiratory Syndrome. N. Engl. J. Med..

[8] Zhang W., Zhao Y., Zhang F., Wang Q., Li T., Liu Z., Wang J., Qin Y., Zhang X., Yan X. (2020). The Use of Anti-Inflammatory Drugs in the Treatment of People with Severe Coronavirus Disease 2019 (COVID-19): The Perspectives of Clinical Immunologists from China. Clin. Immunol..

[9] Wang C., Horby P.W., Hayden F.G., Gao G.F. (2020). A Novel Coronavirus Outbreak of Global Health Concern. Lancet.

[10] Beigel J.H., Tomashek K.M., Dodd L.E., Mehta A.K., Zingman B.S., Kalil A.C., Hohmann E., Chu H.Y., Luetkemeyer A., Kline S. (2020). Remdesivir for the Treatment of Covid-19—Final Report. N. Engl. J. Med..

[11] Cao B., Wang Y., Wen D., Liu W., Wang J., Fan G., Ruan L., Song B., Cai Y., Wei M. (2020). A Trial of Lopinavir-Ritonavir in Adults Hospitalized with Severe Covid-19. N. Engl. J. Med..

[12] Costanzo L., Palumbo F.P., Ardita G., Antignani P.L., Arosio E., Failla G. (2020). Coagulopathy, Thromboembolic Complications, and the Use of Heparin in COVID-19 Pneumonia. J. Vasc. Surg. Venous. Lymphat. Disord..

[13] Choy E.H., de Benedetti F., Takeuchi T., Hashizume M., John M.R., Kishimoto T. (2020). Translating IL-6 Biology into Effective Treatments. Nat. Rev. Rheumatol..

[14] Hu Z., Lv Y., Xu C., Sun W., Chen W., Peng Z., Chen C., Cui X., Jiao D., Cheng C. (2020). Clinical Use of Short-Course and Low-Dose Corticosteroids in Patients with Non-Severe COVID-19 During Pneumonia Progression. Front. Public Health.

[15] Wang K., Tan F., Zhou R., Liu D., Ni Z., Liu J., Luo F. (2020). Therapeutic Response to Corticosteroids in a Critically Ill Patient with COVID-19. Medicine.

[16] Shang L., Zhao J., Hu Y., Du R., Cao B. (2020). On the Use of Corticosteroids for 2019-NCoV Pneumonia. Lancet.

[17] Group T.R.C. (2021). Dexamethasone in Hospitalized Patients with COVID-19. N. Engl. J. Med..

[18] Palumbo A., Chanan-Khan A., Weisel K., Nooka A.K., Masszi T., Beksac M., Spicka I., Hungria V., Munder M., Mateos M.V. (2016). Daratumumab, Bortezomib, and Dexamethasone for Multiple Myeloma. N. Engl. J. Med..

[19] Richardson P.G., Sonneveld P., Schuster M.W., Irwin D., Stadtmauer E.A., Facon T., Harousseau J.-L., Ben-Yehuda D., Lonial S., Goldschmidt H. (2005). Bortezomib or High-Dose Dexamethasone for Relapsed Multiple Myeloma. N. Engl. J. Med..

[20] Plint A.C., Johnson D.W., Patel H., Wiebe N., Correll R., Brant R., Mitton C., Gouin S., Bhatt M., Joubert G. (2009). Epinephrine and Dexamethasone in Children with Bronchiolitis. N. Engl. J. Med..

[21] Hughes H.D., Carroll J.A., Burdick Sanchez N.C., Roberts S.L., Broadway P.R., May N.D., Ballou M.A., Richeson J.T. (2017). Effects of Dexamethasone Treatment and Respiratory Vaccination on Rectal Temperature, Complete Blood Count, and Functional Capacities of Neutrophils in Beef Steers1,2. J. Anim. Sci..

[22] Divari S., de Lucia F., Berio E., Sereno A., Biolatti B., Cannizzo F.T. (2020). Dexamethasone and Prednisolone Treatment in Beef Cattle: Influence on Glycogen Deposition and Gene Expression in the Liver. Domest. Anim. Endocrinol..

[23] Ward S.A., Kirkwood R.N., Plush K.J. (2020). Administering Dexamethasone to Prepartum Sows: Effects on Sow and Piglet Performance. Livest. Sci..

[24] Oulton R.L., Kohn T., Cwiertny D.M. (2010). Pharmaceuticals and Personal Care Products in Effluent Matrices: A Survey of Transformation and Removal during Wastewater Treatment and Implications for Wastewater Management. J. Environ. Monit..

[25] Patel M., Kumar R., Kishor K., Mlsna T., Pittman C.U., Mohan D. (2019). Pharmaceuticals of Emerging Concern in Aquatic Systems: Chemistry, Occurrence, Effects, and Removal Methods. Chem. Rev..

[26] Bu Q., Cao Y., Yu G., He X., Zhang H., Sun J., Yun M., Cao Z. (2020). Identifying Targets of Potential Concern by a Screening Level Ecological Risk Assessment of Human Use Pharmaceuticals in China. Chemosphere.

[27] Ammann A.A., Macikova P., Groh K.J., Schirmer K., Suter M.J.F. (2014). LC-MS/MS Determination of Potential Endocrine Disruptors of Cortico Signalling in Rivers and Wastewaters. Anal. Bioanal. Chem..

[28] Cruz-Morató C., Lucas D., Llorca M., Rodriguez-Mozaz S., Gorga M., Petrovic M., Barceló D., Vicent T., Sarrà M., Marco-Urrea E. (2014). Hospital Wastewater Treatment by Fungal Bioreactor: Removal Efficiency for Pharmaceuticals and Endocrine Disruptor Compounds. Sci. Total Environ..

[29] Ismail N.A.H., Wee S.Y., Kamarulzaman N.H., Aris A.Z. (2019). Quantification of Multi-Classes of Endocrine-Disrupting Compounds in Estuarine Water. Environ. Pollut..

[30] Ismail N.A.H., Wee S.Y., Haron D.E.M., Kamarulzaman N.H., Aris A.Z. (2020). Occurrence of Endocrine Disrupting Compounds in Mariculture Sediment of Pulau Kukup, Johor, Malaysia. Mar. Pollut. Bull..

[31] Tölgyesi Á., Verebey Z., Sharma V.K., Kovacsics L., Fekete J. (2010). Simultaneous Determination of Corticosteroids, Androgens, and Progesterone in River Water by Liquid Chromatography-Tandem Mass Spectrometry. Chemosphere.

[32] Wee S.Y., Aris A.Z., Yusoff F.M., Praveena S.M. (2019). Occurrence and Risk Assessment of Multiclass Endocrine Disrupting Compounds in an Urban Tropical River and a Proposed Risk Management and Monitoring Framework. Sci. Total Environ..

[33] Zhang H.-C., Yu X., Yang W., Peng J., Xu T., Yin D.-Q., Hu X. (2011). MCX Based Solid Phase Extraction Combined with Liquid Chromatography Tandem Mass Spectrometry for the Simultaneous Determination of 31 Endocrine-Disrupting Compounds in Surface Water of Shanghai. J. Chromatogr. B..

[34] Johnson T.L., Tomanek L., Peterson D.G. (2013). A Proteomic Analysis of the Effect of Growth Hormone on Mammary Alveolar Cell-T (MAC-T) Cells in the Presence of Lactogenic Hormones. Domest. Anim. Endocrinol..

[35] Solinas C., Perra L., Aiello M., Migliori E., Petrosillo N. (2020). A Critical Evaluation of Glucocorticoids in the Management of Severe COVID-19. Cytokine Growth Factor Rev..

[36] Keller M.J., Kitsis E.A., Arora S., Chen J.-T., Agarwal S., Ross M.J., Tomer Y., Southern W. (2020). Effect of Systemic Glucocorticoids on Mortality or Mechanical Ventilation in Patients With COVID-19. J. Hosp. Med..

[37] Han Y., Jiang M., Xia D., He L., Lv X., Liao X., Meng J. (2020). COVID-19 in a Patient with Long-Term Use of Glucocorticoids: A Study of a Familial Cluster. Clin. Immunol..

[38] Lu S., Zhou Q., Huang L., Shi Q., Zhao S., Wang Z., Li W., Tang Y., Ma Y., Luo X. (2020). Effectiveness and Safety of Glucocorticoids to Treat COVID-19: A Rapid Review and Meta-Analysis. Ann. Transl. Med..

[39] Stavreva D.A., George A.A., Klausmeyer P., Varticovski L., Sack D., Voss T.C., Schiltz R.L., Blazer V.S., Iwanowicz L.R., Hager G.L. (2012). Prevalent Glucocorticoid and Androgen Activity in US Water Sources. Sci. Rep..

[40] Deblonde T., Cossu-Leguille C., Hartemann P. (2011). Emerging Pollutants in Wastewater: A Review of the Literature. Int. J. Hyg. Environ. Health.

[41] Runnalls T.J., Margiotta-Casaluci L., Kugathas S., Sumpter J.P. (2010). Pharmaceuticals in the Aquatic Environment: Steroids and Anti-Steroids as High Priorities for Research. Hum. Ecol. Risk Assess..

[42] Plotkin B.J., Roose R.J., Erikson Q., Viselli S.M. (2003). Effect of Androgens and Glucocorticoids on Microbial Growth and Antimicrobial Susceptibility. Curr. Microbiol..

[43] Kawabata K., Sugihara K., Sanoh S., Kitamura S., Ohta S. (2013). Photodegradation of Pharmaceuticals in the Aquatic Environment by Sunlight and UV-A, -B and -C Irradiation. Toxicol. Sci..

[44] DellaGreca M., Fiorentino A., Isidori M., Lavorgna M., Previtera L., Rubino M., Temussi F. (2004). Toxicity of Prednisolone, Dexamethasone and Their Photochemical Derivatives on Aquatic Organisms. Chemosphere.

[45] LaLone C.A., Villeneuve D.L., Olmstead A.W., Medlock E.K., Kahl M.D., Jensen K.M., Durhan E.J., Makynen E.A., Blanksma C.A., Cavallin J.E. (2012). Effects of a Glucocorticoid Receptor Agonist, Dexamethasone, on Fathead Minnow Reproduction, Growth, and Development. Environ. Toxicol. Chem..

[46] Chen Q., Jia A., Snyder S.A., Gong Z., Lam S.H. (2016). Glucocorticoid Activity Detected by In Vivo Zebrafish Assay and In Vitro Glucocorticoid Receptor Bioassay at Environmental Relevant Concentrations. Chemosphere.

[47] Jerez-Cepa I., Gorissen M., Mancera J.M., Ruiz-Jarabo I. (2019). What Can We Learn from Glucocorticoid Administration in Fish? Effects of Cortisol and Dexamethasone on Intermediary Metabolism of Gilthead Seabream (*Sparus aurata* L.). Comp. Biochem. Physiol. Part A Mol. Integr. Physiol..

[48] Guo Z., Guo A., Guo Q., Rui M., Zhao Y., Zhang H., Zhu S. (2017). Decomposition of Dexamethasone by Gamma Irradiation: Kinetics, Degradation Mechanisms and Impact on Algae Growth. Chem. Eng. J..

[49] Kugathas S., Sumpter J.P. (2011). Synthetic Glucocorticoids in the Environment: First Results on Their Potential Impacts on Fish. Environ. Sci. Technol..

[50] Sulaiman S., Khamis M., Nir S., Lelario F., Scrano L., Bufo S.A., Karaman R. (2014). Stability and Removal of Dexamethasone Sodium Phosphate from Wastewater Using Modified Clays. Environ. Technol..

[51] Herrero P., Borrull F., Pocurull E., Marcé R.M. (2012). Determination of Glucocorticoids in Sewage and River Waters by Ultra-High Performance Liquid Chromatography–Tandem Mass Spectrometry. J. Chromatogr. A..

[52] Arsand D.R., Kümmerer K., Martins A.F. (2013). Removal of Dexamethasone from Aqueous Solution and Hospital Wastewater by Electrocoagulation. Sci. Total Environ..

[53] Tomlinson E.S., Maggs J.L., Park B.K., Back D.J. (1997). Dexamethasone Metabolism in Vitro: Species Differences. J. Steroid Biochem. Mol. Biol..

[54] Gentile D.M., Tomlinson E.S., Maggs J.L., Park B.K., Back D.J. (1996). Dexamethasone Metabolism by Human Liver in Vitro. Metabolite Identification and Inhibition of 6-Hydroxylation. J. Pharmacol. Exp. Ther..

[55] Pervaiz I., Ahmad S., Mukhtar M.F., Arshad A., Imran M., Mahmood W. (2015). Microbial Biotransformation of Dexamethasone by Bacillus Subtilis (ATCC 6051). Pharm. Chem. J..

[56] Dumasia M.C., Houghton E., Moss M.S., Chakraborty J., Marks V. (1986). The Biotransformation and Urinary Excretion of Dexamethasone in Equine Male Castrates. J. Steroid Biochem. Mol. Biol..

[57] Sirés I., Brillas E. (2012). Remediation of Water Pollution Caused by Pharmaceutical Residues Based on Electrochemical Separation and Degradation Technologies: A Review. Environ. Int..

[58] Calza P., Pelizzetti E., Brussino M., Baiocchi C. (2001). Ion Trap Tandem Mass Spectrometry Study of Dexamethasone Transformation Products on Light Activated TiO_2_ Surface. J. Am. Soc. Mass Spectrom..

[59] Albini A., Fasani E., Albini A., Fasani E. (1998). Photochemistry of drugs: An overview and practical problems. Drugs: Photochem. Photostability.

[60] Chang H., Wan Y., Hu J. (2009). Determination and Source Apportionment of Five Classes of Steroid Hormones in Urban Rivers. Environ. Sci. Technol..

[61] Gong J., Lin C., Xiong X., Chen D., Chen Y., Zhou Y., Wu C., Du Y. (2019). Occurrence, Distribution, and Potential Risks of Environmental Corticosteroids in Surface Waters from the Pearl River Delta, South China. Environ. Pollut..

[62] Zhang J., Luo J., Chang H. (2020). Aerobic Biodegradation of Four Groups of Steroid Hormones in Activated Sludge. J. Chem..

[63] Goh S.X.L., Goh H.A., Lee H.K. (2018). Automation of Ionic Liquid Enhanced Membrane Bag-Assisted-Liquid-Phase Microextraction with Liquid Chromatography-Tandem Mass Spectrometry for Determination of Glucocorticoids in Water. Anal. Chim. Acta.

[64] Mohd Nasir F.A., Praveena S.M., Aris A.Z. (2019). Public Awareness Level and Occurrence of Pharmaceutical Residues in Drinking Water with Potential Health Risk: A Study from Kajang (Malaysia). Ecotoxicol. Environ. Saf..

[65] Praveena S.M., Shaifuddin S.N.M., Sukiman S., Nasir F.A.M., Hanafi Z., Kamarudin N., Ismail T.H.T., Aris A.Z. (2018). Pharmaceuticals Residues in Selected Tropical Surface Water Bodies from Selangor (Malaysia): Occurrence and Potential Risk Assessments. Sci. Total Environ..

[66] Van der Linden S.C., Heringa M.B., Man H.-Y., Sonneveld E., Puijker L.M., Brouwer A., van der Burg B. (2008). Detection of Multiple Hormonal Activities in Wastewater Effluents and Surface Water, Using a Panel of Steroid Receptor CALUX Bioassays. Environ. Sci. Technol..

[67] Schriks M., van Leerdam J.A., van der Linden S.C., van der Burg B., van Wezel A.P., de Voogt P. (2010). High-Resolution Mass Spectrometric Identification and Quantification of Glucocorticoid Compounds in Various Wastewaters in The Netherlands. Environ. Sci. Technol..

[68] Jha R.R., Singh N., Kumari R., Patel D.K. (2017). Ultrasound-Assisted Emulsification Microextraction Based on a Solidified Floating Organic Droplet for the Rapid Determination of 19 Antibiotics as Environmental Pollutants in Hospital Drainage and Gomti River Water. J. Sep. Sci..

[69] Kovalova L., Siegrist H., Singer H., Wittmer A., McArdell C.S. (2012). Hospital Wastewater Treatment by Membrane Bioreactor: Performance and Efficiency for Organic Micropollutant Elimination. Environ. Sci. Technol..

[70] Santos L.H.M.L.M., Gros M., Rodriguez-Mozaz S., Delerue-Matos C., Pena A., Barceló D., Montenegro M.C.B.S.M. (2013). Contribution of Hospital Effluents to the Load of Pharmaceuticals in Urban Wastewaters: Identification of Ecologically Relevant Pharmaceuticals. Sci. Total Environ..

[71] Shen X., Chang H., Sun Y., Wan Y. (2020). Determination and Occurrence of Natural and Synthetic Glucocorticoids in Surface Waters. Environ. Int..

[72] Chang H., Hu J., Shao B. (2007). Occurrence of Natural and Synthetic Glucocorticoids in Sewage Treatment Plants and Receiving River Waters. Environ. Sci. Technol..

[73] Weizel A., Schlüsener M.P., Dierkes G., Ternes T.A. (2018). Occurrence of Glucocorticoids, Mineralocorticoids, and Progestogens in Various Treated Wastewater, Rivers, and Streams. Environ. Sci. Technol..

[74] Praveena S.M., Mohd Rashid M.Z., Mohd Nasir F.A., Wee S.Y., Aris A.Z. (2020). Occurrence, Human Health Risks, and Public Awareness Level of Pharmaceuticals in Tap Water from Putrajaya (Malaysia). Expo. Health.

[75] Mhuka V., Dube S., Nindi M.M. (2020). Occurrence of Pharmaceutical and Personal Care Products (PPCPs) in Wastewater and Receiving Waters in South Africa Using LC-Orbitrap^TM^ MS. Emerg. Contam..

[76] Anumol T., Merel S., Clarke B.O., Snyder S.A. (2013). Ultra High Performance Liquid Chromatography Tandem Mass Spectrometry for Rapid Analysis of Trace Organic Contaminants in Water. Chem. Cent. J..

[77] Piram A., Salvador A., Gauvrit J.-Y., Lanteri P., Faure R. (2008). Development and Optimisation of a Single Extraction Procedure for the LC/MS/MS Analysis of Two Pharmaceutical Classes Residues in Sewage Treatment Plant. Talanta.

[78] Fan Z., Wu S., Chang H., Hu J. (2011). Behaviors of Glucocorticoids, Androgens and Progestogens in a Municipal Sewage Treatment Plant: Comparison to Estrogens. Environ. Sci. Technol..

[79] Liu S., Ying G.-G., Zhao J.-L., Chen F., Yang B., Zhou L.-J., Lai H. (2011). Trace Analysis of 28 Steroids in Surface Water, Wastewater and Sludge Samples by Rapid Resolution Liquid Chromatography-Electrospray Ionization Tandem Mass Spectrometry. J. Chromatogr. A.

[80] Liu S., Ying G.-G., Zhou L.-J., Zhang R.-Q., Chen Z.-F., Lai H.-J. (2012). Steroids in a Typical Swine Farm and Their Release into the Environment. Water Res..

[81] Merlo F., Speltini A., Maraschi F., Sturini M., Profumo A. (2020). HPLC-MS/MS Multiclass Determination of Steroid Hormones in Environmental Waters after Preconcentration on the Carbonaceous Sorbent HA-C@silica. Arab. J. Chem..

[82] Isobe T., Sato K., Joon-Woo K., Tanabe S., Suzuki G., Nakayama K. (2015). Determination of Natural and Synthetic Glucocorticoids in Effluent of Sewage Treatment Plants Using Ultrahigh Performance Liquid Chromatography-Tandem Mass Spectrometry. Environ. Sci. Pollut. Res..

[83] Omar T.F.T., Aris A.Z., Yusoff F.M., Mustafa S. (2019). Risk Assessment of Pharmaceutically Active Compounds (PhACs) in the Klang River Estuary, Malaysia. Environ. Geochem. Health.

[84] Gao G., Li S., Li S., Wang Y., Zhao P., Zhang X., Hou X. (2018). A Combination of Computational-Experimental Study on Metal-Organic Frameworks MIL-53(Al) as Sorbent for Simultaneous Determination of Estrogens and Glucocorticoids in Water and Urine Samples by Dispersive Micro-Solid-Phase Extraction Coupled to UPLC-MS/MS. Talanta.

[85] Xu M., Huang H., Li N., Li F., Wang D., Luo Q. (2019). Occurrence and Ecological Risk of Pharmaceuticals and Personal Care Products (PPCPs) and Pesticides in Typical Surface Watersheds, China. Ecotoxicol. Environ. Saf..

[86] Tenorio-Chávez P., Cerro-López M., Castro-Pastrana L.I., Ramírez-Rodrigues M.M., Orozco-Hernández J.M., Gómez-Oliván L.M. (2020). Effects of Effluent from a Hospital in Mexico on the Embryonic Development of Zebrafish, *Danio Rerio*. Sci. Total Environ..

[87] Bal N., Kumar A., Du J., Nugegoda D. (2017). Multigenerational Effects of Two Glucocorticoids (Prednisolone and Dexamethasone) on Life-History Parameters of Crustacean *Ceriodaphnia Dubia* (Cladocera). Environ. Pollut..

[88] Li X., Ma M., Rene E.R., Ma W., Zhang P. (2019). Changes in Microbial Communities during the Removal of Natural and Synthetic Glucocorticoids in Three Types of River-Based Aquifer Media. Environ. Sci. Pollut. Res..

[89] Dubey D., Dutta V., Shukla V., Kumar N. (2020). Nutrient enrichment in lake ecosystem and its effects on algae and macrophytes. Environmental Concerns and Sustainable Development: Biodiversity, Soil and Waste Management.

[90] Silva S.R., Barbosa F.A.R., Mol M.P.G., Magalhães S.M.S. (2019). Toxicity for Aquatic Organisms of Antiretroviral Tenofovir Disoproxil. J. Environ. Prot. Sci..

[91] Inaba H., Pui C.-H. (2010). Glucocorticoid Use in Acute Lymphoblastic Leukaemia. Lancet Oncol..

[92] Minguez L., Ballandonne C., Rakotomalala C., Dubreule C., Kientz-Bouchart V., Halm-Lemeille M.-P. (2015). Transgenerational Effects of Two Antidepressants (Sertraline and Venlafaxine) on Daphnia Magna Life History Traits. Environ. Sci. Technol..

[93] Pierce A.L., Dickey J.T., Felli L., Swanson P., Dickhoff W.W. (2010). Metabolic Hormones Regulate Basal and Growth Hormone-Dependent Igf2 MRNA Level in Primary Cultured Coho Salmon Hepatocytes: Effects of Insulin, Glucagon, Dexamethasone, and Triiodothyronine. J. Endocrinol..

[94] Lutton B.V., Callard I.P. (2008). Influence of Reproductive Activity, Sex Steroids, and Seasonality on Epigonal Organ Cellular Proliferation in the Skate (*Leucoraja erinacea*). Gen. Comp. Endocrinol..

[95] Li D., Yang X.-L., Zhang S.-J., Lin M., Yu W.-J., Hu K. (2008). Effects of Mammalian CYP3A Inducers on CYP3A-Related Enzyme Activities in Grass Carp (*Ctenopharyngodon idellus*): Possible Implications for the Establishment of a Fish CYP3A Induction Model. Comp. Biochem. Physiol. Part C.

[96] Guiloski I.C., Ribas J.L.C., da Pereira L.S., Neves A.P.P., Silva de Assis H.C. (2015). Effects of Trophic Exposure to Dexamethasone and Diclofenac in Freshwater Fish. Ecotoxicol. Environ. Saf..

[97] Milla S., Wang N., Mandiki S.N.M., Kestemont P. (2009). Corticosteroids: Friends or Foes of Teleost Fish Reproduction?. Comp. Biochem. Physiol. Part A Mol. Integ. Physiol..

[98] Sakalli S., Burkina V., Pilipenko N., Zlabek V., Zamaratskaia G. (2018). In Vitro Effects of Diosmin, Naringenin, Quercetin and Indole-3-Carbinol on Fish Hepatic CYP1A1 in the Presence of Clotrimazole and Dexamethasone. Chemosphere.

[99] Rajashree S., Puvanakrishnan R. (1999). Dexamethasone Induced Alterations in the Levels of Proteases Involved in Blood Pressure Homeostasis and Blood Coagulation in Rats. Mol. Cell. Biochem..

[100] Das C., Thraya M., Vijayan M.M. (2018). Nongenomic Cortisol Signaling in Fish. Gen. Comp. Endocrinol..

[101] Qi X.-Z., Li D.-L., Tu X., Song C.-G., Ling F., Wang G.-X. (2016). Preliminary Study on the Relationship between Dexamethasone and Pathogen Susceptibility on Crucian Carp (*Carassius auratus*). Fish Shellfish Immunol..

[102] Kumari Y., Choo B.K.M., Shaikh M.F., Othman I. (2019). Melatonin Receptor Agonist Piper Betle L. Ameliorates Dexamethasone-induced Early Life Stress in Adult Zebrafish. Exp. Ther. Med..

[103] Jia A., Wu S., Daniels K.D., Snyder S.A. (2016). Balancing the Budget: Accounting for Glucocorticoid Bioactivity and Fate during Water Treatment. Environ. Sci. Technol..

[104] Bain P.A., Williams M., Kumar A. (2014). Assessment of Multiple Hormonal Activities in Wastewater at Different Stages of Treatment. Environ. Toxicol. Chem..

[105] Brnardić I., Ćurković L., Sofilić T., Pavlović D.M., Matijašić G., Grčić I., Rađenović A. (2017). Removal of Heavy Metals and Pharmaceuticals from Contaminated Water Using Waste Sludge-Kinetics and Mechanisms. Clean Soil Air Water.

[106] Pflug N.C., Kupsco A., Kolodziej E.P., Schlenk D., Teesch L.M., Gloer J.B., Cwiertny D.M. (2017). Formation of Bioactive Transformation Products during Glucocorticoid Chlorination. Environ. Sci. Water Res. Technol..

[107] Khetan S.K., Collins T.J. (2007). Human Pharmaceuticals in the Aquatic Environment: A Challenge to Green Chemistry. Chem. Rev..

[108] Metcalfe C., Miao X.-S., Hua W., Letcher R., Servos M., Kümmerer K. (2004). Pharmaceuticals in the Canadian environment. Pharmaceuticals in the Environment.

[109] Verlicchi P., Galletti A., Petrovic M., Barceló D. (2010). Hospital Effluents as a Source of Emerging Pollutants: An Overview of Micropollutants and Sustainable Treatment Options. J. Hydrol..

[110] Luo Y., Guo W., Ngo H.H., Nghiem L.D., Hai F.I., Zhang J., Liang S., Wang X.C. (2014). A Review on the Occurrence of Micropollutants in the Aquatic Environment and Their Fate and Removal during Wastewater Treatment. Sci. Total Environ..

[111] Kenaga E.E., Goring C.A. (1980). Relationship between water solubility, soil sorption, octanol-water partitioning, and concentration of chemicals in biota. Aquatic Toxicology.

[112] Halfon E., Galassi S., Brüggemann R., Provini A. (1996). Selection of Priority Properties to Assess Environmental Hazard of Pesticides. Chemosphere.

[113] Tahar A., Choubert J.-M., Coquery M. (2013). Xenobiotics Removal by Adsorption in the Context of Tertiary Treatment: A Mini Review. Environ. Sci. Pollut. Res..

[114] Pal A., Gin K.Y.-H., Lin A.Y.-C., Reinhard M. (2010). Impacts of Emerging Organic Contaminants on Freshwater Resources: Review of Recent Occurrences, Sources, Fate and Effects. Sci. Total Environ..

[115] Gomes R.L., Scrimshaw M.D., Lester J.N. (2009). Fate of Conjugated Natural and Synthetic Steroid Estrogens in Crude Sewage and Activated Sludge Batch Studies. Environ. Sci. Technol..

[116] Sui Q., Huang J., Deng S., Yu G., Fan Q. (2010). Occurrence and Removal of Pharmaceuticals, Caffeine and DEET in Wastewater Treatment Plants of Beijing, China. Water Res..

[117] Dolar D., Vuković A., Ašperger D., Košutić K. (2011). Effect of Water Matrices on Removal of Veterinary Pharmaceuticals by Nanofiltration and Reverse Osmosis Membranes. J. Environ. Sci..

[118] Forteza M., Galán E., Cornejo J. (1989). Interaction of Dexamethasone and Montmorillonite—Adsorption-Degradation Process. Appl. Clay Sci..

[119] Ghenaatgar A., Tehrani R.M.A., Khadir A. (2019). Photocatalytic Degradation and Mineralization of Dexamethasone Using WO_3_ and ZrO_2_ Nanoparticles: Optimization of Operational Parameters and Kinetic Studies. J. Water Process Eng..

[120] Vadi M., Hossinie N., Shekari Z. (2013). Comparative Study of Adsorption Isotherms Steroidal Anti-Inflammatory Drug Dexamethasone on Carbon Nanotube and Activated Carbon. Orient. J. Chem..

[121] Mohseni S.N., Amooey A.A., Tashakkorian H., Amouei A.I. (2016). Removal of Dexamethasone from Aqueous Solutions Using Modified Clinoptilolite Zeolite (Equilibrium and Kinetic). Int. J. Environ. Sci. Technol..

[122] Vieno N., Tuhkanen T., Kronberg L. (2006). Removal of Pharmaceuticals in Drinking Water Treatment: Effect of Chemical Coagulation. Environ. Technol..

[123] Babu B.R., Venkatesan P., Kanimozhi R., Basha C.A. (2009). Removal of Pharmaceuticals from Wastewater by Electrochemical Oxidation Using Cylindrical Flow Reactor and Optimization of Treatment Conditions. J. Environ. Sci. Health A.

[124] Schäfer A.I., Akanyeti I., Semião A.J.C. (2011). Micropollutant Sorption to Membrane Polymers: A Review of Mechanisms for Estrogens. Adv. Colloid Interface Sci..

[125] Sorell T.L. (2016). Approaches to the Development of Human Health Toxicity Values for Active Pharmaceutical Ingredients in the Environment. AAPS J..

[126] Besha A.T., Gebreyohannes A.Y., Tufa R.A., Bekele D.N., Curcio E., Giorno L. (2017). Removal of Emerging Micropollutants by Activated Sludge Process and Membrane Bioreactors and the Effects of Micropollutants on Membrane Fouling: A Review. J. Environ. Chem. Eng..

[127] Ioannou-Ttofa L., Michael-Kordatou I., Fattas S.C., Eusebio A., Ribeiro B., Rusan M., Amer A.R.B., Zuraiqi S., Waismand M., Linder C. (2017). Treatment Efficiency and Economic Feasibility of Biological Oxidation, Membrane Filtration and Separation Processes, and Advanced Oxidation for the Purification and Valorization of Olive Mill Wastewater. Water Res..

[128] Goswami L., Vinoth Kumar R., Borah S.N., Arul Manikandan N., Pakshirajan K., Pugazhenthi G. (2018). Membrane Bioreactor and Integrated Membrane Bioreactor Systems for Micropollutant Removal from Wastewater: A Review. J. Water Process Eng..

[129] Grandclément C., Seyssiecq I., Piram A., Wong-Wah-Chung P., Vanot G., Tiliacos N., Roche N., Doumenq P. (2017). From the Conventional Biological Wastewater Treatment to Hybrid Processes, the Evaluation of Organic Micropollutant Removal: A Review. Water Res..

[130] Wardenier N., Liu Z., Nikiforov A., van Hulle S.W.H., Leys C. (2019). Micropollutant Elimination by O_3_, UV and Plasma-Based AOPs: An Evaluation of Treatment and Energy Costs. Chemosphere.

[131] Prieto-Rodríguez L., Oller I., Klamerth N., Agüera A., Rodríguez E.M., Malato S. (2013). Application of Solar AOPs and Ozonation for Elimination of Micropollutants in Municipal Wastewater Treatment Plant Effluents. Water Res..

[132] Pazoki M., Parsa M., Farhadpour R. (2016). Removal of the Hormones Dexamethasone (DXM) by Ag Doped on TiO_2_ Photocatalysis. J. Environ. Chem. Eng..

[133] Vestel J.S., Hong J.-Y., Meng Q., Naumann B.D., Robson M.G., Sargent E.V. (2017). The Endocrine Disruption Potential of Betamethasone Using Japanese Medaka as a Fish Model. Hum. Ecol. Risk Assess..

[134] Hernando M.D., Mezcua M., Fernández-Alba A.R., Barceló D. (2006). Environmental Risk Assessment of Pharmaceutical Residues in Wastewater Effluents, Surface Waters and Sediments. Talanta.

[135] Al Aukidy M., Verlicchi P., Voulvoulis N. (2014). A Framework for the Assessment of the Environmental Risk Posed by Pharmaceuticals Originating from Hospital Effluents. Sci. Total Environ..

[136] Ankley G.T., Bennett R.S., Erickson R.J., Hoff D.J., Hornung M.W., Johnson R.D., Mount D.R., Nichols J.W., Russom C.L., Schmieder P.K. (2010). Adverse Outcome Pathways: A Conceptual Framework to Support Ecotoxicology Research and Risk Assessment. Environ. Toxicol. Chem..

[137] Musee N. (2018). Environmental Risk Assessment of Triclosan and Triclocarban from Personal Care Products in South Africa. Environ. Pollut..

