# Supplementary material for: Occurrence, Fate, Effects, and Risks of Dexamethasone: Ecological Implications Post-COVID-19
Source: Int J Environ Res Public Health. 2021 Oct 27;18(21):11291. doi: 10.3390/ijerph182111291 (PMC8583091; doi:10.3390/ijerph182111291)
Supplement: Supplementary file 1 [file ijerph-18-11291-s001.zip › ijerph-1380987-supplementary.pdf]

Supplementary information

Occurrence, fate, effects, and risks of dexamethasone: ecological implications post-COVID-19

Ndeke Musee[1,*], Lemme P. Kebaabetswe[2], Shepherd Tichapondwa[3], Gosaitse Tubatsi[3], Ntombikayise Mahaye[1], Samuel K. Leareng[1], Philiswa Nomngongo[4]

[1] Emerging Contaminants Ecological Risk Assessment (ECERA) Group, Department of Chemical Engineering, University of Pretoria, Pretoria South Africa

[2] Department of Biological Sciences and Biotechnology, Botswana International University of Science and Technology, Palapye, Botswana

[3] Water Utilization and Environmental Engineering Division, Department of Chemical Engineering, University of Pretoria, Pretoria, 0002, South Africa

[4] DSI/NRF SARChI Chair: Nanotechnology for Water, Department of Chemical Sciences, University of Johannesburg, Doornfontein, 2028, South Africa

* Corresponding author (N Musee), Email address: ndeke.musee@up.ac.za or museen2012@gmail.com

Table S1. The ecotoxicological effects of DEXA on organisms at different levels of organization

DEXA Properties	Organism type	Organism	Exposure concentration	Exposure conditions	Duration	Effects studied (Endpoints)	Effects observed	NOEC, LC_{50}/EC_{50}	Refs
DEXA	Microbial community	Bacteria	500 ng/L	Aquifer media, Beijing Chaobai River (BJ), Hebei Hutuo River (HB), and Tianjin Duliujian River water samples	50 d	Microbial community changes	DEXA completely degraded, with removal rates varying due to microbial diversity and composition	NR	[1]
Irradiated and non-irradiated DEXA	*Photobacterium phosphorium*	Bacteria	NR	2% NaCl solution	15 min	Bioluminescence test	The solution containing DEXA was more toxic than DEXA following irradiation for 24 h	N.D before irradiation, EC_{50} =133.80 mg/L following irradiation	[2]

DEXA and TPs	*Pseudokirchenerie lla subcapitata*	Algae	NR	ISO procedure 8692, under continuous illumination of 8000 lux at 25 ± 1 °C	72 h	Growth inhibition	DEXA had no effect on algal growth, while TPs inhibited algal growth	EC_{50} = 12.15, 40.75, > 100 mg/L, for TP11, TP12 and DEXA, respectively	[3]
Non-irradiated DEXA<br><br>4 000 gamma-irradiated DEXA	*Microcystis flos-aquae*<br><br>*Scenedesmus obliquus*	Algae	25, 50 and 100 mg/L	BG 11 medium, light/dark cycle of 12 h/12 h, at 25 ± 1 °C	14 d	-Algal growth<br><br>-Chlorophyll-a content	-Non-irradiated DEXA induced concentration-dependent algal growth promotion, and increase chlorophyll-a content for both algal species<br><br>- For radiated DEXA, chlorophyll-a content of *M. flos-aquae* increased by 4.27%, but decrease by 25.65% for *S. obliquus*	NR	[4]

DEXA and TPs	*Brachionus calyciflorus*	Rotifer	NR	Moderately hard medium EPA, Hardness was 80–100 mg/l $CaCO_3$, dissolved oxygen content was at least 90%, 25 ± 1 °C in the dark	24 h	Mortality	TPs were more toxic than parent DEXA	LC_{50}= 13.20, 44.66 and 48.22 mg/L, respectively for TP 11, TP 12 and DEXA	[3]
DEXA and TPs	*Thamnocephalus platyurus*	Crustacean	NR	Moderately hard EPA medium, 25 ± 1 °C in the dark	24 h	Mortality	TPs were more toxic than parent DEXA	LC_{50}= 20.9, 30.52 and 60.11 mg/L, respectively for TP 11, TP 12 and DEXA	[3]
DEXA and TPs	*Daphnia magna*	Crustacean	NR	Aerated synthetic reconstituted, hardness 250 mg/l expressed as	24 h	Immobilization	TPs were more toxic than parent DEXA	EC_{50}= 10.88, 17.82, and 48.30,mg/L, respectively,	[3]

				$CaCO_3$, 20 ± 1 °C in the dark freshwater				for TP 11,TP 12 and DEXA	
DEXA and TPs	*Ceriodaphnia dubia*	Crustacean	NR	Synthetic reconstituted aerated hard ISO medium, hardness 250 mg/l as $CaCO_3$, at 25 ± 1 °C with a 16:8-h light: dark cycle (500 lux).	7 d	Population growth inhibition	TPs were more toxic than parent DEXA	EC_{50} = 0.05, 0.13, and 0.06 mg/L for DEXA, TP 11 and TP 12, respectively	[3]
DEXA	*Ceriodaphnia dubia*	Crustacean	0.05 to 3.2 mg/L	Moderately hard reconstituted water	48 h	Immobilization	Immobility was observed	EC_{50} = 0.75 mg/L	[5]
DEXA	*Ceriodaphnia dubia*	Crustacean	1.95-125 µg/L	Moderately hard reconstituted water	NR	Multigenerational chronic bioassays	Significant reduction in reproduction was observed at the	NR	[5]

							lowest concentration of 3.9 µg/L		
DEXA	Rainbow trout *(Oncorhynchus mykiss)*, (hepatic microsomes)	Fish	0.39, 19.62 mg/L	Freshwater 14±1°C and DEXA prepared in DMSO and METOH and exposure at 21°C	10 min	Fish CYP1A-enzyme activity (EROD)	No effect on EROD activity	NR	[6]
DEXA with indole-3-carbinol	Rainbow trout (hepatic microsones)	Fish	0.39, 19.62 mg/L	Freshwater 14±1°C and DEXA prepared in DMSO and METOH and exposure at 21°C	10 min	Fish CYP1A-enzyme activity (EROD)	EROD activity declined	NR	[6]
Betamethasone	Japanese medaka	Fish	0.01, 0.1, 1.0 µg/L used in two-generation	Continuous flow through diluter system/water	0-133 d	-Secondary sexual characterisation,<br>-vitellogenin expression	-Wet weight was low<br>-Endocrine related disruptions developed	NOEC = 0.1 µg/L	[7]

			fish full life cycle				-Lifetime exposures resulted in low reproductive success,<br>-3-fold increase in vitellogenin expression in males at 1.0 µg/L		
DEXA	Adult *Hoplias malabaricus*	Fish	0, 0.03, 0.3, or 3 µg/kg	Filtered and dechlorinated tap water.<br>Trophic exposure light/dark cycle of 12h/12h at 24±1 ºC	30 d	- Oxidative stress<br>- Biotransformation in liver and gonads<br>- Endocrine disruption	No effect of EROD activity	NR	[8]
DEXA	*Sparus aurata L.*	Fish	800 mol/m³	DEXA administered through food pellets	35 d	- Intermediary metabolism<br>- Growth	- Lower growth rates<br>- Lower body mass		[9]

- High hepatosomatic index (HIS)
- Reduced plasma cortisol levels
- Reduced TAG
- High acid levels
- Increased oxidation in both liver and muscles
- High GPDH (glucose metabolism)
- Enhanced hepatic storage
- Higher GLDH and LDH activities in muscles
- Plasma osmolality higher

DEXA	*Damio rerio* larvae and adult male fish	Fish	50/500 mg/L<br>5/50 mg/L	E3 medium for 120 h at 28 ± 0.5 ºC<br>12 h at 29 ± 1.0 ºC	4 d	Glucocorticoid responsive genes	- Unregulated expression of *pepck, biap2* in zebrafish larvae<br>- *Baiap2, prx*, and *mmp2* unregulated in adult zebrafish	EC_{50} =1.8nM	[10]
DEXA	Male and female juvenile zebrafish	Fish	-0.1, 0.2, 4 and 15.8 mg/L	Egg water at 28°C<br>14 h light/10 h darkness cycle, water and air at 4 °C and 23 °C, respectively<br>Eggs:13 h light/11 h dark at 28 °C	All exposed from 1 h-48 h and 48 h – 96 h post infections	- dynamics of glucocorticoid uptake by zebrafish chlorinated embryos	-uptake dependent on developmental stages of the embryo	NR	[11]

DEXA	-Rainbow trout<br><br>-Mummichog killifish (*Fundulus heteroclitus*)	Fish	50, 100 mg/kg (trout)<br>100 mg/kg (killifish)	Killfish at 19-20°C on recirculating sea water<br><br>Trout at 12-15°C in flow-through water	48 h	Metabolic rates	- Killifish exhibited higher metabolic rates<br>- DEXA induced 3-cyano-7-ethoxycoumarin metabolism	NR	[12]
DEXA	Fathead Minnow *(Pimephales promelas)*	Fish	62.5, 125, 250, 500, 1000 µg/L	Dimethylformamide or METOH<br><br>16 h light/8 h dark at 28 ^{o}C ± 1 ^{o}C	28 d	Survival and growth	-decreased survival<br><br>-growth not impacted	LC_{50}=254 µg/L<br><br>NOEC= 254 µg/L, and LOEC=577 µg/L	[13]
DEXA	Fathead Minnow	Fish	0.1, 50, 500 µg/L	Continuous flow-thru exposures conducted by pumping diluted DEXA at 25±1°C with a photoperiod of 16	-21 d reproduction study<br><br>-29 d embryo-larvae	-reproductive toxicity and early life effects	-500 µg/L exposure at 21 d caused:<br><br>-reduction in fecundity, female plasma estradiol, increase in plasma	NR	[14]

				h light and 8 h dark.			vitellogenin protein levels<br>-500 µg/L exposure at 29 d resulted in deformed gill opercula, reduction in weight and length		
DEXA	Crucian Carp (*Cyprinus carpio*)	Fish	20 mg/L	Dechlorinated water at 25°C	1, 2, 4, 6, 8, 10 d	-effects on regeneration process in caudal fin	-smaller fins<br>-regeneration delayed but no apparent inhibition	NR	[15]
DEXA	Rainbow trout	Fish	0.39, 3.93, 19.62 and 39.25 mg/L	Freshwater	5, 10, 30 min	-Inhibitory effects of Dexa on 3 CYP450 mediated activities; (EROD, CYP1A)<br>(BFC-OD, CYP3A)<br>(PNPH, CYP2E1)	-no inhibitory potency on any enzyme	NR	[16]

DEXA	Rainbow trout (juvenile)	Fish	3, 30 300 and 3000 ng/L	Freshwater	21 and 42 d	-effects on CYP450 system on fish	-no change in CYP450 activities<br>-induction of the CYP3A protein observed at the lowest concentration	NR	[16]
DEXA	Crucian carp (*Carassius auratus*)	Fish	200 µg/kg	NR	0, 3, 6 and 9 d	- effects on the immune response of DEXA treated fish infected with *Aeromonas hydrophila*	- fish exposed to DEXA and then infected with a pathogen, had higher mortality<br>-Immune gene expression *il-1β, cxcl-8*, *tnfα*, and *crp* down-regulated	NR	[17]
DEXA	Sterlet (*Acipenser ruthensus L.*)		0.8 mg/ml	Aerated tanks at 16-18°C, fish exposed to DEXA in Ringers solution	0, 1, 3, 7, 14 and 21 d	- effects of dexa on the oxidative processes in the immunocompete	- activation of lipid peroxidation	NR	[18]

						nt organs (liver, kidney and spleen)	-decrease in antioxidant activity		
DEXA	Adult Zebrafish	Fish	2 and 20 mg/l	Freshwater. 14 h light and 10 h dark.	24 h	- effects of stress on behaviour, melatonin and stress-related gene expression (*nfkb)*	-anxious behaviour, lower level of melatonin, higher mRNA expression of *nfkb*	NR	[19]
DEXA and *Piper betle L.* (PB) leaf extract and Melatonin	Adult Zebrafish	Fish	-Melatonin (10mg/kg)<br>- *Piper betle L.*(30mg/kg) exposed to fish after DEXA treatment	Freshwater. 14 h light and 10 h dark.	24 h	- effects on behaviour and expression of<br>- levels of melatonin related genes (*MTI, MT2, aanat1, aanat2*) and stress-related (*nfkb*) gene	-PB enhanced sleep behaviour in fish<br>-*aanat2* suppression on PB, melatonin and DEXA treated fish<br>-*MT2* increased expression on PB, melatonin and DEXA	NR	[19]

							-no change in the *MT1* and *aanat1* expression.<br>-suppressed *nfkb* mRNA expression		
DEXA	Crucian Carp	Fish	1mg/L	Dechlorinated tap water at 25-26°C.<br>14h light: 10h dark;	35 d	- assessing immunomodulatory effects of chemical pollutants on fish using DEXA followed by exposure to *Aeromonas salmonicida*	-100% mortality on bacteria-infected fish exposed to DEXA<br>- lower leukocytes and haemolytic complement activity	NR	[20]

NR-not reported

Table S2. Removal of DEXA during wastewater treatment in WWTPs with techniques utilized across geographical regions globally in selected studies.

Location	Technology	Water Type (Environmental matrix)	Influent conc.	Effluent conc.	%Removal	Sludge	Reference
Italy	Adsorption<br>Modified clay / GAC	Dexamethasone sodium phosphate (DSP) from Wastewater	100 mg/L		GAC (35.7%), Clay (< LQL) (Outlet)		[21]
Brazil	Electrocoagulation	Hospital wastewater	0.1 mg/L		38%		[22]<br>[23]
Croatia	Adsorption<br>Waste<br>Sludge	Industrial Wastewater	80 mg/L		80%		[24]
India	Electro coagulation	Synthetic wastewater	1 400 mg/L		86%		[25]<br>[4]

Iran	Photocatalysis ZrO_2 and WO_3	Synthetic wastewater	5 - 50 mg/L		20 – 100%	[4]
Iran	Photocatalysis Ag/TiO_2 visible light	Synthetic wastewater	15 mg/L		30 – 70%	[26]
Iran	Sono-nanocatalysis	Synthetic wastewater	15 – 45 mg/L		60 – 90 %	[27]
Croatia	Membranes NF and RO	Synthetic wastewater	10 mg/L		> 90%	[28] [29]
Switzerland	Membrane bioreactor	Hospital Wastewater	147 ng/L	ND	100%	[30]
USA	Ozonation	Synthetic wastewater	2 mg/L		60%	[31]
Italy	Photocatalysis TiO_2–UV light	Synthetic wastewater	10 mg/L		100%	[32]

	Adsorption Modified clinoptilolite zeolite		5 – 40 mg/L		35 – 80%		[33], [119]
Spain	Adsorption Montmorillonite	Synthetic wastewater	50 mg/L		90.6%		[21]
China	Gamma irradiation	Synthetic wastewater	2 – 50 mg/L		95 – 100%		[34]
	Lagoons	Swine wastewater	260 ng/L	37.8 ng/L	85%	35.0 ng/g	[35]
	Biological WWT	WW influent (Huiyang WWTP)	22.6 ng/L	ND*	100%	ND	[36]
		WW influent (Meihu WWTP)	3.8 ng/L	ND*	100%	ND	
USA	AOP and Membranes Ozone, UV, RO, MF	WW effluent	0.11–0.16 ng/L	< 0.06 ng/L		-	[37]

Australia	Biological WWT including constructed wetlands	Municipal wastewater		60 ng/L DEX equiv. (SBR)<br>51–85 ng/LDEX equiv.	Sequence batch reactor (SBR) below LQL<br>Increases after chlorination<br>70 ng/L DEX equiv.		[23]
China	Biological Wastewater Treatment	Municipal wastewater		0.81 ng/L	0.03 ng/L	<0.02 ng/g	[38]

*Conc.: concentration; LQL: lower quantification limit

References

1. Li, X.; Ma, M.; Rene, E.R.; Ma, W.; Zhang, P. Changes in Microbial Communities during the Removal of Natural and Synthetic Glucocorticoids in Three Types of River-Based Aquifer Media. *Environ Sci Pollut Res* **2019**, *26*, 33953–33962, doi:10.1007/s11356-018-2748-x.
2. Kawabata, K.; Sugihara, K.; Sanoh, S.; Kitamura, S.; Ohta, S. Photodegradation of Pharmaceuticals in the Aquatic Environment by Sunlight and UV-A, -B and -C Irradiation. *The Journal of Toxicological Sciences* **2013**, *38*, 215–223, doi:10.2131/jts.38.215.
3. DellaGreca, M.; Fiorentino, A.; Isidori, M.; Lavorgna, M.; Previtera, L.; Rubino, M.; Temussi, F. Toxicity of Prednisolone, Dexamethasone and Their Photochemical Derivatives on Aquatic Organisms. *Chemosphere* **2004**, *54*, 629–637, doi:10.1016/j.chemosphere.2003.09.008.
4. Guo, Z.; Guo, A.; Guo, Q.; Rui, M.; Zhao, Y.; Zhang, H.; Zhu, S. Decomposition of Dexamethasone by Gamma Irradiation: Kinetics, Degradation Mechanisms and Impact on Algae Growth. *Chemical Engineering Journal* **2017**, *307*, 722–728, doi:10.1016/j.cej.2016.08.138.
5. Bal, N.; Kumar, A.; Du, J.; Nugegoda, D. Multigenerational Effects of Two Glucocorticoids (Prednisolone and Dexamethasone) on Life-History Parameters of Crustacean *Ceriodaphnia Dubia* (Cladocera). *Environmental Pollution* **2017**, *225*, 569–578, doi:10.1016/j.envpol.2017.03.024.
6. Sakalli, S.; Burkina, V.; Pilipenko, N.; Zlabek, V.; Zamaratskaia, G. In Vitro Effects of Diosmin, Naringenin, Quercetin and Indole-3-Carbinol on Fish Hepatic CYP1A1 in the Presence of Clotrimazole and Dexamethasone. *Chemosphere* **2018**, *192*, 105–112, doi:10.1016/j.chemosphere.2017.10.106.
7. Vestel, J.S.; Hong, J.-Y.; Meng, Q.; Naumann, B.D.; Robson, M.G.; Sargent, E.V. The Endocrine Disruption Potential of Betamethasone Using Japanese Medaka as a Fish Model. *Human and Ecological Risk Assessment: An International Journal* **2017**, *23*, 879–894, doi:10.1080/10807039.2017.1292841.
8. Guiloski, I.C.; Ribas, J.L.C.; Pereira, L. da S.; Neves, A.P.P.; Silva de Assis, H.C. Effects of Trophic Exposure to Dexamethasone and Diclofenac in Freshwater Fish. *Ecotoxicology and Environmental Safety* **2015**, *114*, 204–211, doi:10.1016/j.ecoenv.2014.11.020.
9. Jerez-Cepa, I.; Gorissen, M.; Mancera, J.M.; Ruiz-Jarabo, I. What Can We Learn from Glucocorticoid Administration in Fish? Effects of Cortisol and Dexamethasone on Intermediary Metabolism of Gilthead Seabream (Sparus Aurata L.). *Comparative Biochemistry and Physiology Part A: Molecular & Integrative Physiology* **2019**, *231*, 1–10, doi:10.1016/j.cbpa.2019.01.010.
10. Chen, Q.; Jia, A.; Snyder, S.A.; Gong, Z.; Lam, S.H. Glucocorticoid Activity Detected by in Vivo Zebrafish Assay and in Vitro Glucocorticoid Receptor Bioassay at Environmental Relevant Concentrations. *Chemosphere* **2016**, *144*, 1162–1169, doi:10.1016/j.chemosphere.2015.09.089.
11. Steenbergen, P.J.; Bardine, N.; Sharif, F. Kinetics of Glucocorticoid Exposure in Developing Zebrafish: A Tracer Study. *Chemosphere* **2017**, *183*, 147–155, doi:10.1016/j.chemosphere.2017.05.059.

12. Smith, E.M.; Wilson, J.Y. Assessment of Cytochrome P450 Fluorometric Substrates with Rainbow Trout and Killifish Exposed to Dexamethasone, Pregnenolone-16α-Carbonitrile, Rifampicin, and β-Naphthoflavone. *Aquatic Toxicology* **2010**, *97*, 324–333, doi:10.1016/j.aquatox.2010.01.005.
13. Overturf, M.D.; Overturf, C.L.; Baxter, D.; Hala, D.N.; Constantine, L.; Venables, B.; Huggett, D.B. Early Life-Stage Toxicity of Eight Pharmaceuticals to the Fathead Minnow, Pimephales Promelas. *Arch Environ Contam Toxicol* **2012**, *62*, 455–464, doi:10.1007/s00244-011-9723-6.
14. LaLone, C.A.; Villeneuve, D.L.; Olmstead, A.W.; Medlock, E.K.; Kahl, M.D.; Jensen, K.M.; Durhan, E.J.; Makynen, E.A.; Blanksma, C.A.; Cavallin, J.E.; et al. Effects of a Glucocorticoid Receptor Agonist, Dexamethasone, on Fathead Minnow Reproduction, Growth, and Development. *Environmental Toxicology and Chemistry* **2012**, *31*, 611–622, doi:10.1002/etc.1729.
15. Ochandio, B.S.; Bechara, I.J.; Parise-Maltempi, P.P. Dexamethasone Action on Caudal Fin Regeneration of Carp *Cyprinus Carpio* (Linnaeus, 1758). *Braz. J. Biol.* **2015**, *75*, 442–450, doi:10.1590/1519-6984.16813.
16. Burkina, V.; Zlabek, V.; Zamaratskaia, G. Clotrimazole, but Not Dexamethasone, Is a Potent in Vitro Inhibitor of Cytochrome P450 Isoforms CYP1A and CYP3A in Rainbow Trout. *Chemosphere* **2013**, *92*, 1099–1104, doi:10.1016/j.chemosphere.2013.01.050.
17. Qi, X.-Z.; Li, D.-L.; Tu, X.; Song, C.-G.; Ling, F.; Wang, G.-X. Preliminary Study on the Relationship between Dexamethasone and Pathogen Susceptibility on Crucian Carp (Carassius Auratus). *Fish & Shellfish Immunology* **2016**, *59*, 18–24, doi:10.1016/j.fsi.2016.10.017.
18. Mikryakov, D.V.; Mikryakov, V.R.; Silkina, N.I. Effect of Dexamethasone on Oxidative Processes in the Immunocompetent Organs of Sterlet Acipenser Ruthenus L. *Inland Water Biol* **2014**, *7*, 397–400, doi:10.1134/S1995082914040117.
19. Kumari, Y.; Choo, B.K.M.; Shaikh, M.F.; Othman, I. Melatonin Receptor Agonist Piper Betle L. Ameliorates Dexamethasone-induced Early Life Stress in Adult Zebrafish. *Experimental and Therapeutic Medicine* **2019**, *18*, 1407–1416, doi:10.3892/etm.2019.7685.
20. Nakayama, K.; Yamashita, R.; Kitamura, S.-I. Use of Common Carp (Cyprinus Carpio) and Aeromonas Salmonicida for Detection of Immunomodulatory Effects of Chemicals on Fish. *Marine Pollution Bulletin* **2017**, *124*, 710–713, doi:10.1016/j.marpolbul.2016.12.060.
21. Pazoki, M.; Parsa, M.; Farhadpour, R. Removal of the Hormones Dexamethasone (DXM) by Ag Doped on TiO_2 Photocatalysis. *Journal of Environmental Chemical Engineering* **2016**, *4*, 4426–4434, doi:10.1016/j.jece.2016.09.034.
22. Jia, A.; Wu, S.; Daniels, K.D.; Snyder, S.A. Balancing the Budget: Accounting for Glucocorticoid Bioactivity and Fate during Water Treatment. *Environmental Science & Technology* **2016**, *50*, 2870–2880.
23. Pflug, N.C.; Kupsco, A.; Kolodziej, E.P.; Schlenk, D.; Teesch, L.M.; Gloer, J.B.; Cwiertny, D.M. Formation of Bioactive Transformation Products during Glucocorticoid Chlorination. *Environ. Sci.: Water Res. Technol.* **2017**, *3*, 450–461, doi:10.1039/C7EW00033B.

24. Cuerda-Correa, E.M.; Alexandre-Franco, M.F.; Fernández-González, C. Advanced Oxidation Processes for the Removal of Antibiotics from Water. An Overview. *Water* **2020**, *12*, 102, doi:10.3390/w12010102.
25. Tahar, A.; Choubert, J.-M.; Coquery, M. Xenobiotics Removal by Adsorption in the Context of Tertiary Treatment: A Mini Review. *Environ Sci Pollut Res* **2013**, *20*, 5085–5095, doi:10.1007/s11356-013-1754-2.
26. Boxall, A.B.; Rudd, M.A.; Brooks, B.W.; Caldwell, D.J.; Choi, K.; Hickmann, S.; Innes, E.; Ostapyk, K.; Staveley, J.P.; Verslycke, T. Pharmaceuticals and Personal Care Products in the Environment: What Are the Big Questions? *Environmental health perspectives* **2012**, *120*, 1221–1229.
27. Sorell, T.L. Approaches to the Development of Human Health Toxicity Values for Active Pharmaceutical Ingredients in the Environment. *AAPS J* **2016**, *18*, 92–101, doi:10.1208/s12248-015-9818-5.
28. Sulaiman, S.; Khamis, M.; Nir, S.; Lelario, F.; Scrano, L.; Bufo, S.A.; Karaman, R. Stability and Removal of Dexamethasone Sodium Phosphate from Wastewater Using Modified Clays. *Environmental Technology* **2014**, *35*, 1945–1955, doi:10.1080/09593330.2014.888097.
29. Arsand, D.R.; Kümmerer, K.; Martins, A.F. Removal of Dexamethasone from Aqueous Solution and Hospital Wastewater by Electrocoagulation. *Science of the Total Environment* **2013**, *443*, 351–357.
30. Garcia-Segura, S.; Eiband, M.M.S.G.; de Melo, J.V.; Martínez-Huitle, C.A. Electrocoagulation and Advanced Electrocoagulation Processes: A General Review about the Fundamentals, Emerging Applications and Its Association with Other Technologies. *Journal of Electroanalytical Chemistry* **2017**, *801*, 267–299, doi:10.1016/j.jelechem.2017.07.047.
31. Brnardić, I.; Ćurković, L.; Sofilić, T.; Pavlović, D.M.; Matijašić, G.; Grčić, I.; Rađenović, A. Removal of Heavy Metals and Pharmaceuticals From Contaminated Water Using Waste Sludge–Kinetics and Mechanisms. *CLEAN–Soil, Air, Water* **2017**, *45*, 1600509.
32. Babu, B.R.; Venkatesan, P.; Kanimozhi, R.; Basha, C.A. Removal of Pharmaceuticals from Wastewater by Electrochemical Oxidation Using Cylindrical Flow Reactor and Optimization of Treatment Conditions. *Journal of Environmental Science and Health, Part A* **2009**, *44*, 985–994, doi:10.1080/10934520902996880.
33. Sirés, I.; Brillas, E. Remediation of Water Pollution Caused by Pharmaceutical Residues Based on Electrochemical Separation and Degradation Technologies: A Review. *Environment International* **2012**, *40*, 212–229, doi:10.1016/j.envint.2011.07.012.
34. Rahmani, H.; Rahmani, K.; Rahmani, A.; Zare, M.-R. Removal of Dexamethasone from Aqueous Solutions Using Sono-Nanocatalysis Process. *Research Journal of Environmental Sciences* **2015**, *9*, 320–331, doi:10.3923/rjes.2015.320.331.
35. Dolar, D.; Vuković, A.; Ašperger, D.; Košutić, K. Effect of Water Matrices on Removal of Veterinary Pharmaceuticals by Nanofiltration and Reverse Osmosis Membranes. *Journal of Environmental Sciences* **2011**, *23*, 1299–1307.
36. Dolar, D.; Košutić, K.; Ašperger, D. Influence of Adsorption of Pharmaceuticals onto RO/NF Membranes on Their Removal from Water. *Water, Air, & Soil Pollution* **2013**, *224*, 1377.

37. Kovalova, L.; Siegrist, H.; Singer, H.; Wittmer, A.; McArdell, C.S. Hospital Wastewater Treatment by Membrane Bioreactor: Performance and Efficiency for Organic Micropollutant Elimination. *Environmental science & technology* **2012**, *46*, 1536–1545.
38. Calza, P.; Pelizzetti, E.; Brussino, M.; Baiocchi, C. Ion Trap Tandem Mass Spectrometry Study of Dexamethasone Transformation Products on Light Activated TiO_2 Surface. *J Am Soc Mass Spectrom* **2001**, *12*, 1286–1295, doi:10.1016/S1044-0305(01)00319-1.
